# The Kv1.3 blocker PAP‑1 alleviates psoriasis‑like dermatitis in rats via suppressing inflammation and angiogenesis

**DOI:** 10.1038/s42003-026-10718-7

**Published:** 2026-07-20

**Authors:** Feng Yao, Yong Zhang, Yanmin Zheng, Renpeng Zhou, Tianqi Liu, Lulu Wang, Gengxin Wang, Qiuxia Yu, Demeng Sun, Sanling Liu, Changlin Tian, Wei Hu

**Affiliations:** 1 Department of Clinical Pharmacology, The Second Affiliated Hospital of Anhui Medical University, Hefei, Anhui China; 2 Division of Life Sciences and Medicine, Joint Center for Biological Analytical Chemistry, Anhui Engineering Laboratory of Peptide Drug, University of Science and Technology of China, Hefei, Anhui China; 3 Anhui Provincial Key Laboratory of High Field Magnetic Resonance Imaging, High Magnetic Field Laboratory, Hefei Institute of Physical Science, Chinese Academy of Sciences, Hefei, Anhui China; 4 Beijing Life Science Academy, Beijing, China; 5 School of Chemistry and Chemical Engineering, Zhangjiang Institute for Advanced Study, Shanghai Jiao Tong University, Shanghai, China; 6 School of Biomedical Engineering, Suzhou Institute for Advanced Research, University of Science and Technology of China, Suzhou, Jiangsu China

**Keywords:** Cell invasion, Psoriasis, Cryoelectron microscopy

## Abstract

Psoriasis is a chronic inflammatory skin disorder driven by epidermal hyperplasia, immune infiltration, and pathological angiogenesis. PAP‑1, a selective Kv1.3 channel blocker, exhibits therapeutic potential, though its underlying mechanisms remain incompletely understood. Here, we show that both preventive and therapeutic administration of PAP‑1 markedly attenuates imiquimod (IMQ)-induced psoriasis‑like skin lesions and reduces splenomegaly in male rats. RNA sequencing revealed that PAP-1 downregulated genes associated with inflammation and angiogenesis, accompanied by decreased levels of pro-inflammatory cytokines and vascular markers. PAP-1 also inhibited VEGF-A-induced HUVEC proliferation, migration, invasion, and tube formation. Mechanistically, PAP-1 not only blocked Kv1.3 currents, but also suppressed Kv1.3 expression in the skin of IMQ-induced psoriasis-like male rats and VEGF-A-induced HUVECs. Cryo-EM structural analysis revealed that PAP-1 binds within the central cavity of Kv1.3, beneath the selectivity filter, in an inactivated state. These results establish that preventive and therapeutic PAP-1 alleviates psoriasis-like dermatitis via dual anti-inflammatory and anti-angiogenic mechanisms, supporting its further therapeutic development.

## Abstract (web summary)

Combining transcriptomic, functional, and cryo-EM structural analyses, the selective Kv1.3 blocker PAP-1 is identified as a promising therapeutic strategy for psoriasis by demonstrating potent anti-inflammatory and anti-angiogenic effects in preclinical models.

## Introduction

Psoriasis is a chronic inflammatory skin disorder characterized by abnormal keratinocyte proliferation, infiltrating inflammatory cells, and increased angiogenesis in the dermis^1,2^. Abnormal keratinocyte proliferation and immunological dysfunction are central to the pathology of this disorder, which has been extensively studied. However, before the onset of psoriatic skin lesions, microvascular changes such as vascular dilation and elongation, increased vascular permeability, and tortuosity can be observed histologically. This suggests that increased angiogenesis is also a key factor in the pathogenesis of psoriasis^3,4^. During the development of psoriasis, numerous immune cells and abnormally activated keratinocytes produce large amounts of cytokines and mediators that promote angiogenesis, such as interleukin (IL)-17A and vascular endothelial growth factor (VEGF)-A^5^. IL-17A not only directly mediates angiogenesis but also enhances the expression of other angiogenic factors, including VEGF-A^6^. These studies indicate a crosstalk between angiogenesis-related inflammatory cytokines and angiogenic mediators, leading to the exacerbation of the inflammatory response and the aggravation of angiogenesis in psoriatic skin lesions.

The Kv1.3 potassium ion channel (encoded by *KCNA3*) plays a critical role in subsets of T and B lymphocytes, and therapeutic targeting of Kv1.3 has shown benefits in several significant autoimmune diseases, including psoriasis, rheumatoid arthritis, contact dermatitis, and multiple sclerosis^7,8^. Currently, several small-molecule inhibitors and specific peptides targeting Kv1.3 have successfully reached clinical trials as potential therapeutics for autoimmune diseases. ShK-186 (dalatazide), a synthetic analog of sea anemone toxin ShK developed through chemical modification, has successfully advanced to clinical trials for the treatment of autoimmune diseases^9^. Additionally, YR-001 is a highly selective small molecule inhibitor of Kv1.3, used for the treatment of atopic dermatitis and psoriasis. YR-001 topical ointment was approved by the US FDA for clinical trials in January 2023, and the US phase I clinical trial was successfully completed at the end of October, showing good safety and tolerability in all subjects. Moreover, DES-7114 is a highly selective oral small molecule inhibitor of Kv1.3 that has demonstrated efficacy in preclinical models of several chronic inflammatory and autoimmune diseases, including ulcerative colitis, Crohn’s disease, and atopic dermatitis. A phase I clinical trial of DES-7114 in healthy individuals was completed in early 2022. Therefore, targeting Kv1.3 holds promising potential for the treatment of autoimmune diseases. 5-(4-Phenoxybutoxy) psoralen (PAP-1) has been established as a potent and selective small‑molecule blocker of Kv1.3^10^. Previous work demonstrated that PAP‑1 suppresses the proliferation and pro‑inflammatory cytokine (e.g., IL‑2, IFN‑γ) production of T cells isolated from psoriatic lesions^11^. Importantly, topical application of a 2% PAP‑1 ointment was shown to reduce epidermal thickness by approximately 50% in a SCID mouse‑psoriasis xenograft model, highlighting its direct therapeutic potential at the disease site^11^. However, while these studies validate the local efficacy of PAP‑1, its effects on systemic inflammatory responses and disease‑associated angiogenesis - key pathological features of psoriasis - remain unexplored.

Here, we employed an imiquimod (IMQ)‑induced psoriasis‑like rat model and administered PAP‑1 via intraperitoneal injection. This approach allowed us to evaluate not only its impact on local skin pathology but also its influence on systemic manifestations and angiogenic processes. Using transcriptomic profiling, functional assays in endothelial cells, and structural biology, we aimed to elucidate the multi‑faceted mechanisms through which PAP‑1 alleviates psoriatic inflammation and angiogenesis, thereby expanding the therapeutic rationale for Kv1.3 inhibition in psoriasis.

## Results

### PAP‑1 alleviates skin lesions in IMQ-induced psoriasis-like male rats

**Fig. 1:**
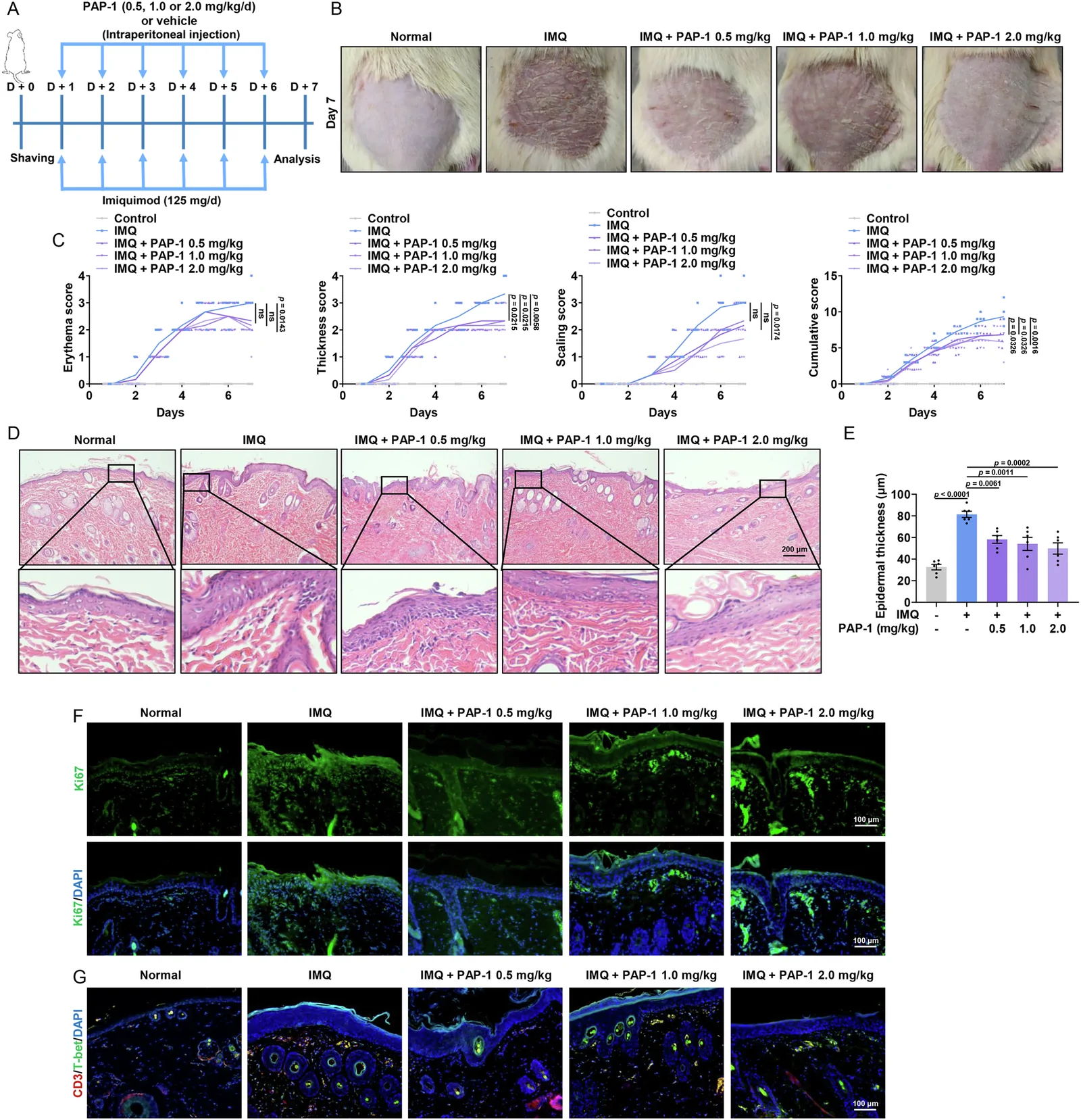
Preventive effects of PAP‑1 in IMQ-induced psoriasis-like male rats. **A** Schematic diagram of the preventive intervention protocol. Male rats received daily topical application of IMQ cream on shaved dorsal skin for six consecutive days, combined with intraperitoneal injection of PAP‑1 (0.5, 1.0, or 2.0 mg/kg/d) or vehicle. **B** Representative photographs of dorsal skin lesions in each group on day 7. **C** Daily erythema, thickness, scaling, and cumulative PASI scores in all groups (*n* = 6). **D** Representative H&E‑stained skin sections (*n* = 6). Scale bar = 200 μm. **E** Quantitative analysis of epidermal thickness (*n* = 6). **F** Representative immunofluorescence images showing Ki67 (green) expression in skin tissues (*n* = 4). Scale bar =  100 μm. **G** Representative immunofluorescence images showing CD3 (red) and T‑bet (green) expression in skin tissues (*n* = 4). Scale bar = 100 μm. *p* < 0.05 was considered statistically significant; ns indicates non‑significant.

**Fig. 2:**
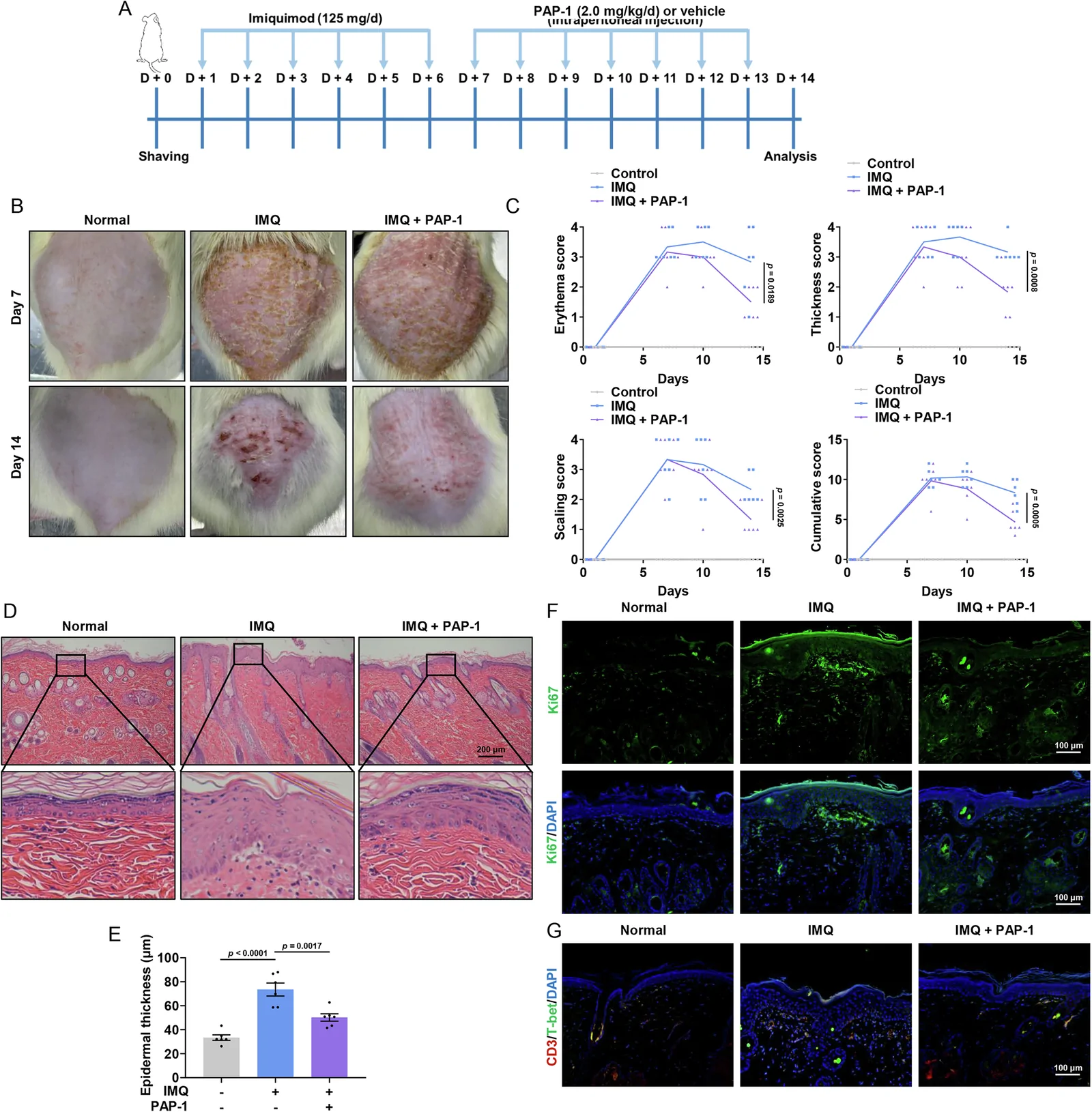
Therapeutic effects of PAP‑1 in IMQ‑induced psoriasis‑like male rats. **A** Schematic diagram of the therapeutic treatment protocol. Male rats were topically treated with IMQ cream for six consecutive days, followed by seven days of intraperitoneal injection of PAP‑1 (2.0 mg/kg/d) or vehicle. **B** Representative photographs of dorsal skin lesions in each group on day 7 and day 14. **C** Erythema, thickness, scaling, and cumulative PASI scores on day 1, 7, 10, and 14 (*n* = 6). **D** Representative images of H&E staining of shaved back skin samples from each rat group (*n* = 6). Scale bar = 200 μm. **E** Quantitative analysis of epidermal thickness (*n* = 6). **F** Representative immunofluorescence images showing Ki67 (green) expression in skin tissues (*n* = 4). Scale bar = 100 μm. **G** Representative immunofluorescence images showing CD3 (red) and T‑bet (green) expression in skin tissues (*n* = 4). Scale bar = 100 μm. *p* < 0.05 was considered statistically significant.

To evaluate the preventive potential of PAP‑1, we established an IMQ‑induced psoriasis‑like male rat model with or without concurrent PAP‑1 intervention. As outlined in Fig. 1A, male rats received daily topical application of IMQ cream on shaved dorsal skin for six consecutive days, combined with intraperitoneal injection of PAP‑1 (0.5, 1.0, or 2.0 mg/kg/d) or vehicle. Compared with the normal group, IMQ‑treated rats developed typical psoriatic lesions, including erythema, skin thickening, and scaling (Fig. 1B). PAP‑1 administration at all tested doses significantly ameliorated these dermatitis symptoms. Additionally, all Psoriasis Area and Severity Index (PASI) parameters, including erythema, thickness, and scaling, were evaluated, and cumulative scores were calculated. On day 7, PAP‑1 at 0.5 and 1.0 mg/kg/d significantly reduced thickness and cumulative PASI scores, while the 2.0 mg/kg/d dose markedly lowered all individual scores and the total score (Fig. 1C). These results indicate that preventive PAP‑1 treatment attenuates the severity of psoriatic lesions in IMQ-induced male rats. H&E staining showed that intervention with 0.5, 1.0, and 2.0 mg/kg/d PAP‑1 alleviated epidermal hyperplasia and significantly decreased epidermal thickness compared to the IMQ‑treated group (Fig. 1D, E). Immunofluorescence analysis indicated that preventive PAP‑1 treatment decreased Ki67 expression (Fig. 1F) and suppressed infiltration of CD3^+^T‑bet^+^ cells (Fig. 1G), suggesting inhibition of keratinocyte proliferation and Th1‑type inflammation. To assess the therapeutic efficacy of PAP‑1, a separate cohort of IMQ‑induced psoriasis‑like male rats received PAP‑1 treatment after lesion establishment. As illustrated in Fig. 2A, male rats were topically treated with IMQ cream for six days, followed by seven days of intraperitoneal PAP‑1 (2.0 mg/kg/d) or vehicle. PAP‑1 treatment visibly improved psoriatic skin lesions (Fig. 2B). On day 14, all PASI parameters as well as the cumulative score were significantly lower in the PAP‑1 treatment group compared to the IMQ group (Fig. 2C). H&E staining showed that PAP‑1 treatment alleviated epidermal hyperplasia and significantly decreased epidermal thickness compared to the IMQ‑treated group (Fig. 2D, E). Additionally, PAP‑1 treatment downregulated Ki67 expression (Fig. 2F) and suppressed the infiltration of CD3^+^T‑bet^+^ cells in the skin (Fig. 2G), indicating inhibition of keratinocyte hyperproliferation and Th1‑type inflammation. In summary, both preventive and therapeutic administration of PAP‑1 effectively ameliorated IMQ‑induced psoriasis‑like dermatitis in male rats, associated with reduced epidermal hyperplasia, decreased cellular proliferation, and suppression of Th1‑mediated inflammation.

Safety is a crucial factor in drug development. To evaluate the potential toxicity of intraperitoneally administered PAP‑1, we performed H&E staining on major organs (heart, liver, spleen, lung, and kidney). Histopathological analysis showed that neither preventive (0.5, 1.0, or 2.0 mg/kg/d) nor therapeutic (2.0 mg/kg/d) PAP‑1 treatment induced significant pathological changes compared with the IMQ group (Supplementary Fig. 1A and B). Body weight did not differ significantly among groups (Supplementary Fig. 1C and D). While IMQ markedly increased serum creatinine levels, preventive and therapeutic PAP‑1 administration did not affect this parameter (Supplementary Fig. 1E and F). In contrast, preventive and therapeutic treatment with 2.0 mg/kg/d PAP‑1 significantly suppressed the IMQ‑induced elevation of serum AST and ALT levels (Supplementary Fig. 1G–J). Together, these results indicate that intraperitoneal injection of PAP‑1 is well tolerated and relatively safe in male rats during the 7‑day treatment period.

### Non‑invasive MRI tracked psoriasis progression and PAP‑1 prevention

**Fig. 3:**
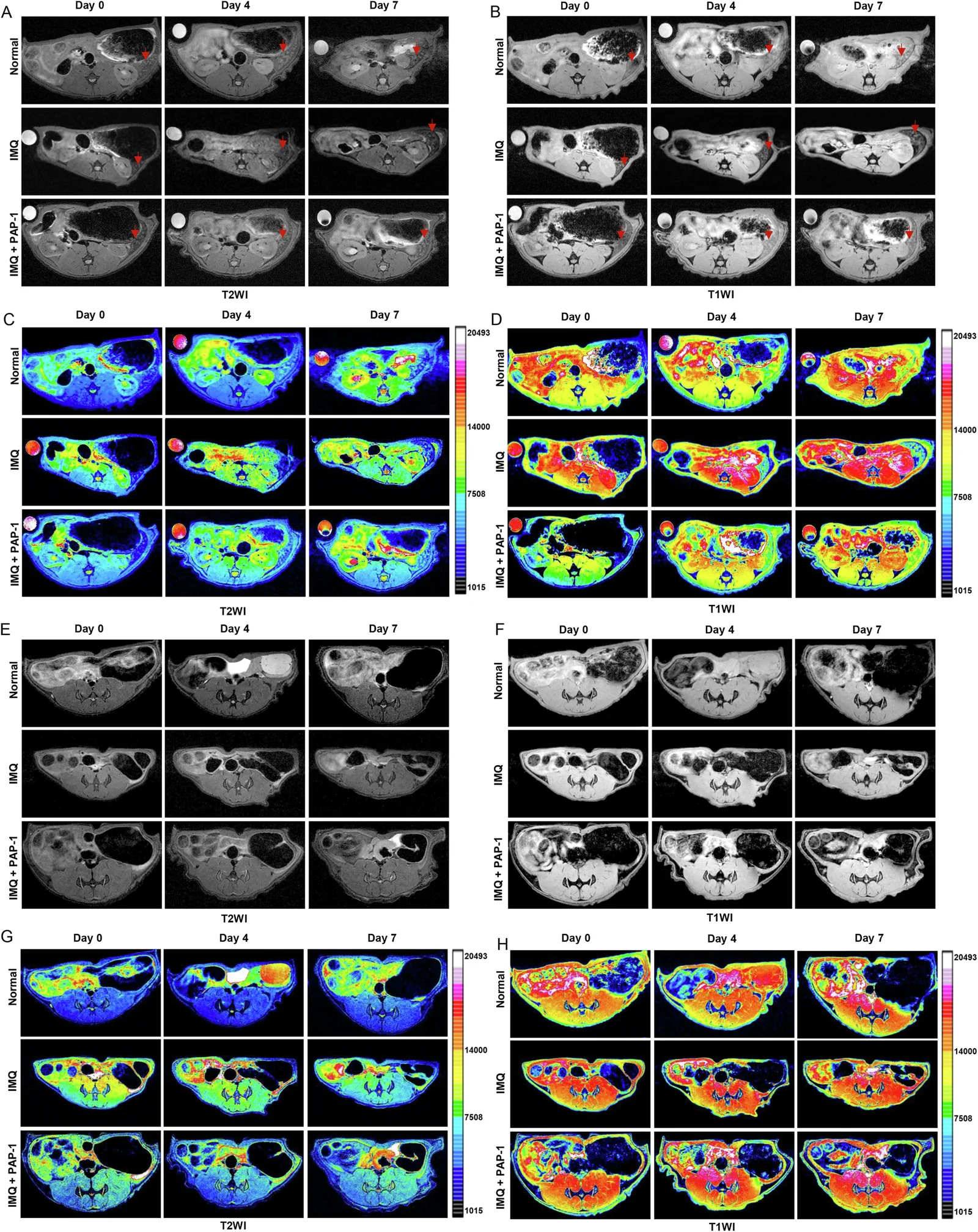
Non‑invasive MRI tracked psoriasis progression and PAP‑1 prevention. **A**, **B** T2-weighted imaging (T2WI) and T1-weighted imaging (T1WI) of the spleen region showed the cross-sectional area of spleen. **C**, **D** T2WI and T1WI signal distribution in the spleen region. **E**, **F** T2WI and T1WI of the shaved area showed the cross-sectional scan. **G**, **H** T2WI and T1WI signal distribution in the shaved area.

At 0, 4, and 7 days, cross-sectional MRI images of the spleen and the dorsal skin (shaved area) were acquired at a consistent vertebral level in preventive and concurrent intervention male rats. This level was selected based on the clear visibility of the vertebral body and paravertebral muscles, which served as stable anatomical landmarks. Comparative analysis of T2WI and T1WI structural images and signal distribution maps revealed different trends across groups. Specifically, the IMQ group showed a progressive enlargement of splenic cross-sectional area over time, whereas no obvious change was observed in either the normal control group or the preventive 1.0 mg/kg/d PAP-1 treatment group (Fig. 3A, B). We further processed the images of T2WI and T1WI, normalized the image signals, obtained pseudo-color maps of signal distribution. On the T2WI images, the cross-sectional signal distribution results of the spleen area of the male rats were analyzed and found that that there was a loss of signal in the IMQ group at the time points of 4 and 7 days. Although there was a certain loss of cross-sectional signals in the spleen area in the preventive PAP-1 treatment group, the missing part also partially recovered with the increase of treatment time. However, there was no obvious change in the spleen signal distribution of male rats in the control group (Fig. 3C). On the T1WI images, the cross-sectional signal distribution results of the spleen area of the three groups of male rats showed no obvious changes at different scanning time points (Fig. 3D). In addition, we also acquired the images of T2WI and T1WI in the shaved area of male rats. The skin thickness of the shaved area of the IMQ and PAP-1 treatment group were obviously thickened and folded. However, there was no obvious change in the cross-sectional scan of the shaving area of the normal male rats (Fig. 3E, F). On the T2WI images, the cross-sectional signal distribution results of the shaved area of male rats found that there was a loss of signal in the IMQ group at the time points of 4 and 7 ds. Consistent with the spleen results, although there was a certain loss of cross-sectional signals in the shaved area in the preventive PAP-1 treatment group, the missing part also partially recovered with the increase of treatment time (Fig. 3G). On the T1WI images, the cross-sectional signal distribution results of the shaved area of the three groups of male rats showed no obvious changes at different scanning time points (Fig. 3H). Non-invasive MRI confirmed reduced splenomegaly and skin damage in PAP-1-treated male rats.

### PAP-1 treatment suppresses inflammatory response and angiogenesis in IMQ-induced psoriasis-like male rats

**Fig. 4:**
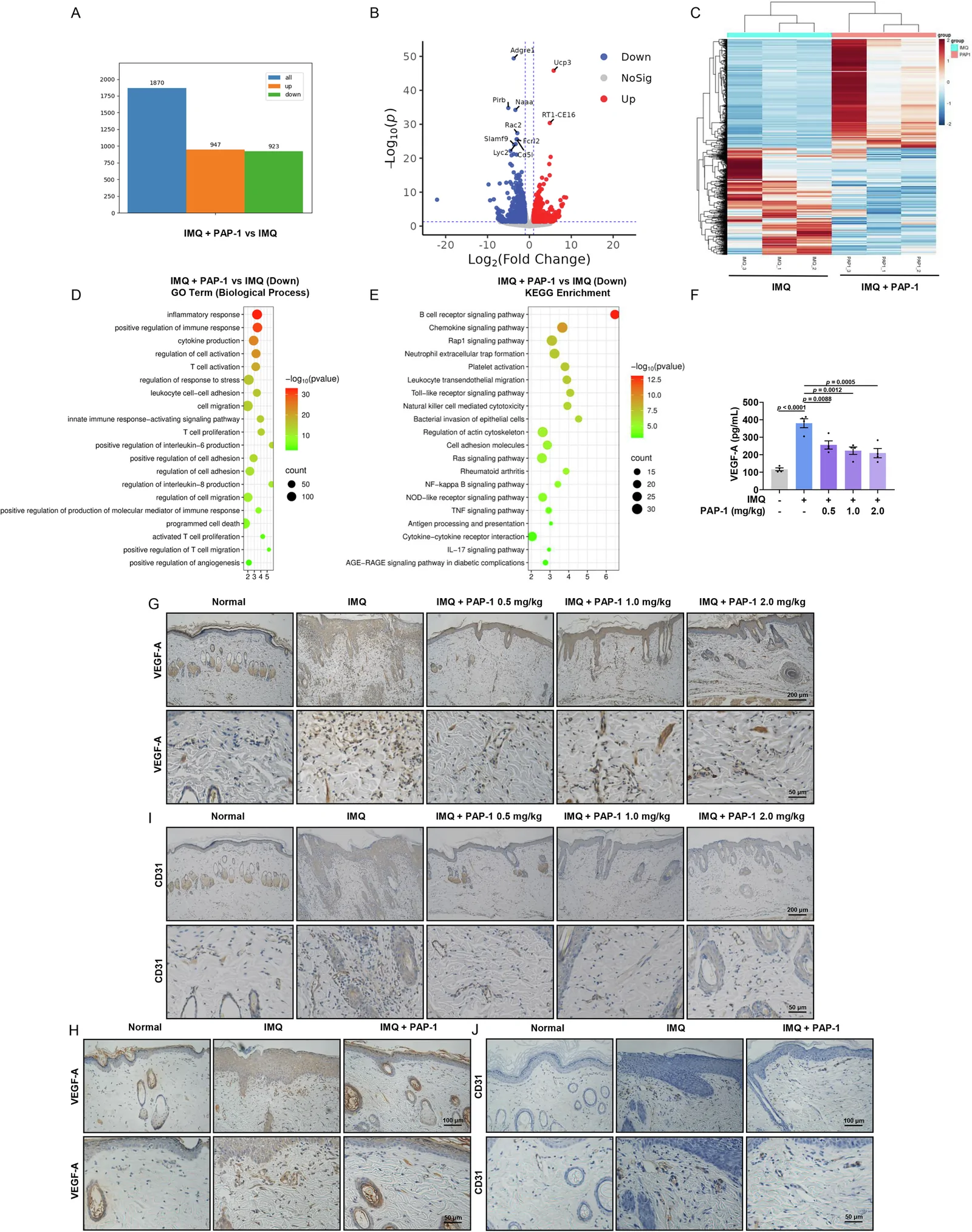
PAP-1 treatment suppresses angiogenesis in IMQ-induced psoriasis-like male rats. **A** PAP-1 treatment upregulated 947 genes and downregulated 923 genes (|log_2_FoldChange | > 1, *p* < 0.05). **B** Volcano plot depicting downregulated (blue) and upregulated (red) DEGs between the 1.0 mg/kg/d PAP-1 treated and untreated IMQ-induced rat skins. **C** Heatmap illustrating the expression patterns of DEGs. **D** Classification of downregulated DEGs based on their GO terms. **E** KEGG enrichment analyses highlighting signaling pathways associated with the downregulated DEGs. **F** Serum VEGF‑A levels in male rats receiving preventive PAP‑1 treatment (*n* = 4 male rats/group). **G** Representative immunohistochemical staining of VEGF‑A expression in skin tissues from preventive PAP-1 treatment. Scale bars = 200 μm and 50 μm. **H** Representative immunohistochemical staining of VEGF‑A expression in skin tissues from therapeutic PAP-1 treatment. Scale bars = 100 μm and 50 μm. **I** Representative immunohistochemical staining of CD31 expression in skin tissues from preventive PAP-1 treatment. Scale bars = 200 μm and 50 μm. **J** Representative immunohistochemical staining of CD31 expression in skin tissues from therapeutic PAP-1 treatment. Scale bars = 100 μm and 50  μm. *p* < 0.05 was considered statistically significant.

To further elucidate the molecular mechanisms underlying the alleviation of IMQ‑induced psoriasis‑like dermatitis by PAP‑1, we performed RNA sequencing on skin tissues from the preventive model, comparing male rats treated with 1.0 mg/kg/d PAP‑1 with untreated IMQ‑induced male rats. Compared to the IMQ group, 1870 genes were significantly altered, with 947 genes upregulated and 923 genes downregulated in the PAP-1 treatment group (|log_2_FoldChange | > 1, significant *p* value < 0.05) (Fig. 4A). Figure 4B presents a volcano plot illustrating changes in differentially expressed genes (DEGs) following PAP-1 treatment, and Fig. 4C shows the corresponding heat map. GO enrichment analysis of the downregulated DEGs revealed enrichment in biological processes including inflammatory response, positive regulation of immune response, cytokine production, regulation of cell activation, T cell activation, regulation of response to stress, T cell proliferation, positive regulation of interleukin-6 production, regulation of interleukin-8 production, positive regulation of angiogenesis (Fig. 4D). KEGG enrichment analysis revealed that these downregulated DEGs were involved in multiple signaling pathways, including the B cell receptor signaling pathway, Rap1 signaling pathway, Neutrophil extracellular trap formation, Toll-like receptor signaling pathway, Cell adhesion molecules, Ras signaling pathway, NF-kappa B signaling pathway, TNF signaling pathway, IL-17 signaling pathway (Fig. 4E). Overall, these findings suggest that PAP-1 may mitigate inflammatory responses and angiogenesis, thereby alleviating IMQ-induced psoriasis-like dermatitis in male rats.

The spleen index serves as a reliable indicator of systemic inflammatory responses. Our results demonstrate that intraperitoneal administration of PAP‑1, whether given preventively (0.5, 1.0, or 2.0 mg/kg/d) or therapeutically (2.0 mg/kg/d), significantly reduced spleen size in IMQ‑induced psoriasis‑like male rats (Supplementary Fig. 2A–F). Correspondingly, while IMQ treatment markedly increased the spleen index, both preventive and therapeutic PAP‑1 administration effectively reversed this elevation (Supplementary Fig. 2G and H). In addition, preventive PAP‑1 treatment significantly lowered serum levels of key inflammatory cytokines, including IL‑17 A, IL‑33, and IL‑6 (Supplementary Fig. 2I–K). In lesional skin, the mRNA expression of these cytokines was notably upregulated in IMQ‑treated male rats compared with normal controls, and preventive PAP‑1 treatment significantly suppressed their expression (Supplementary Fig. 2L–N). Taken together, these findings demonstrate that PAP‑1 alleviates both systemic and local cutaneous inflammation, supporting its therapeutic potential in IMQ‑induced psoriasis‑like conditions.

Angiogenesis plays a crucial role in the pathogenesis of psoriasis, with VEGF-A and CD31 serving as markers of vascular endothelial cells^5,12^. Our results showed that preventive treatment with 0.5, 1.0, and 2.0 mg/kg/d PAP-1 significantly reduced the concentrations of VEGF-A in the serum of IMQ-induced male rats (Fig. 4F). Furthermore, we examined the effect of PAP‑1 on dermal microvascular changes by assessing VEGF‑A and CD31 expression in lesional skin. Compared with the IMQ group, both preventive and therapeutic PAP‑1 treatment led to decreased expression of VEGF‑A and CD31 (Fig. 4G–J). These findings highlight the anti‑angiogenic activity of PAP‑1 in alleviating IMQ‑induced psoriasis‑like skin lesions.

### PAP-1 inhibits angiogenesis in VEGF-A-induced HUVECs

**Fig. 5:**
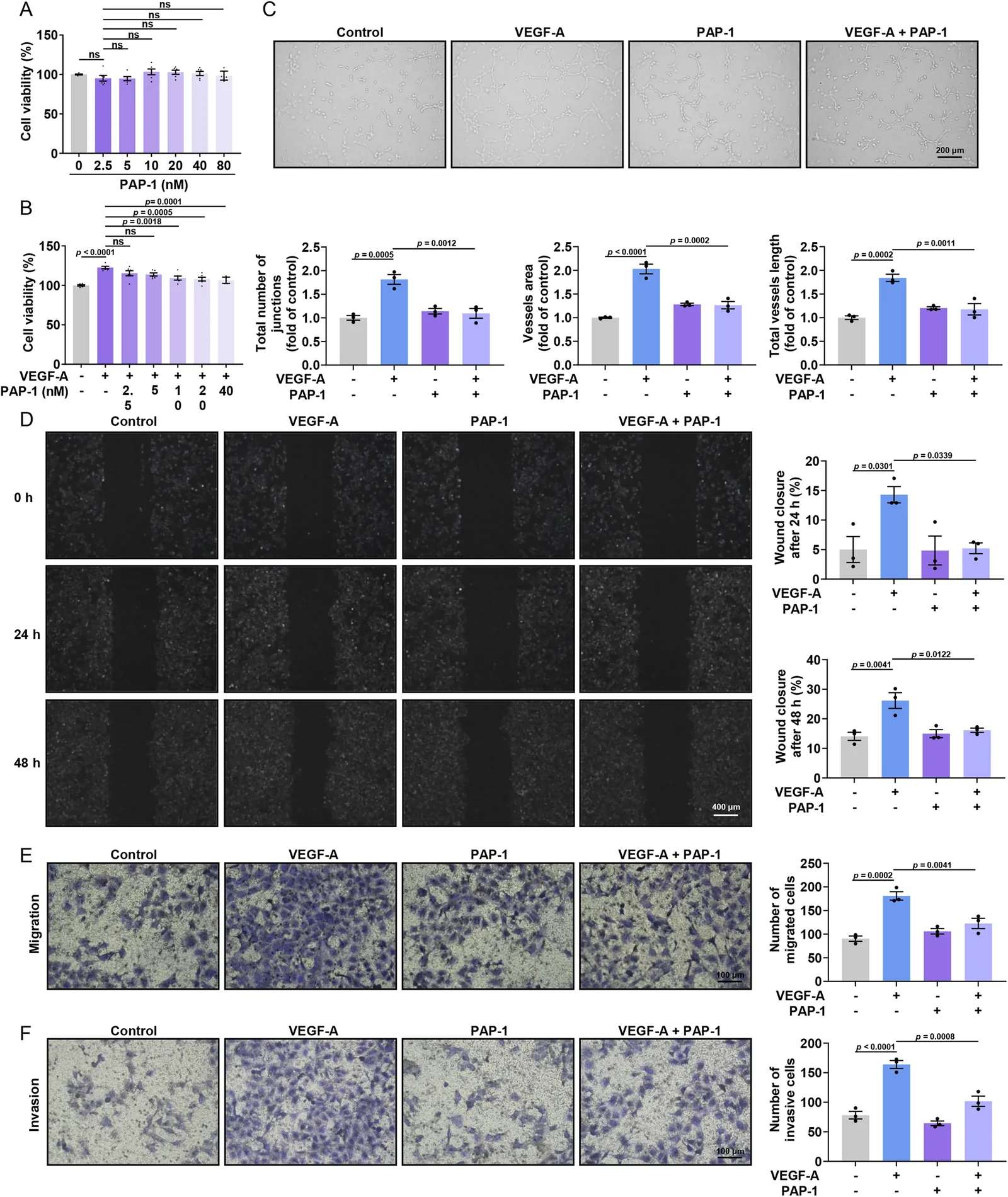
PAP-1 inhibits angiogenesis in VEGF-A-induced HUVECs. **A** HUVEC viability was assessed following incubation with varying concentrations of PAP-1 (0, 2.5, 5, 10, 20, 40, or 80 nM) for 48 h. **B** HUVECs were treated with PAP-1 (2.5, 5, 10, 20, or 40 nM) in the presence of VEGF-A (10 ng/mL) for 48 h, and cell viability was measured using CCK-8 assay. **C** VEGF-A-induced HUVECs were seeded on Matrigel with vehicle or PAP-1 treatment to assess tube formation ability. Scale bar = 200 μm. **D** Representative images and quantification of the wound closure rate. Scale bar = 400 μm. **E**, **F** Representative images and quantification of cell migration and invasion. Scale bar = 200 μm. Data are presented as mean ± SEM, *n* = 3. *p* < 0.05 was considered statistically significant; ns indicates non‑significant.

To assess the potential toxicity of PAP-1, human umbilical vein endothelial cells (HUVECs) were treated with various concentrations (2.5, 5, 10, 20, 40, or 80 nM) for 48 h, and cell viability was evaluated using the CCK-8 assay. No significant difference in cell viability was observed between the PAP-1-treated groups and the control group (Fig. 5A). However, cell viability was significantly reduced in VEGF-A-induced HUVECs treated with 10, 20, and 40 nM PAP-1 for 48 h (Fig. 5B). Additionally, 10 nM PAP-1 markedly impaired the ability of HUVECs to form capillary-like structures on Matrigel in VEGF-A-induced conditions (Fig. 5C). Furthermore, PAP-1 significantly attenuated the wound healing rate of VEGF-A-induced HUVECs (Fig. 5D). Moreover, transwell migration and invasion assays demonstrated that PAP-1 reduced the number of migrated and invasive HUVECs induced by VEGF-A through the membrane (Fig. 5E, F). These findings indicate that PAP-1 inhibits VEGF-A-induced tube formation in HUVECs by suppressing cell proliferation, migration, and invasion.

### PAP-1 inhibits Kv1.3 expression in skin of IMQ-induced male rats and VEGF-A-induced HUVECs

**Fig. 6:**
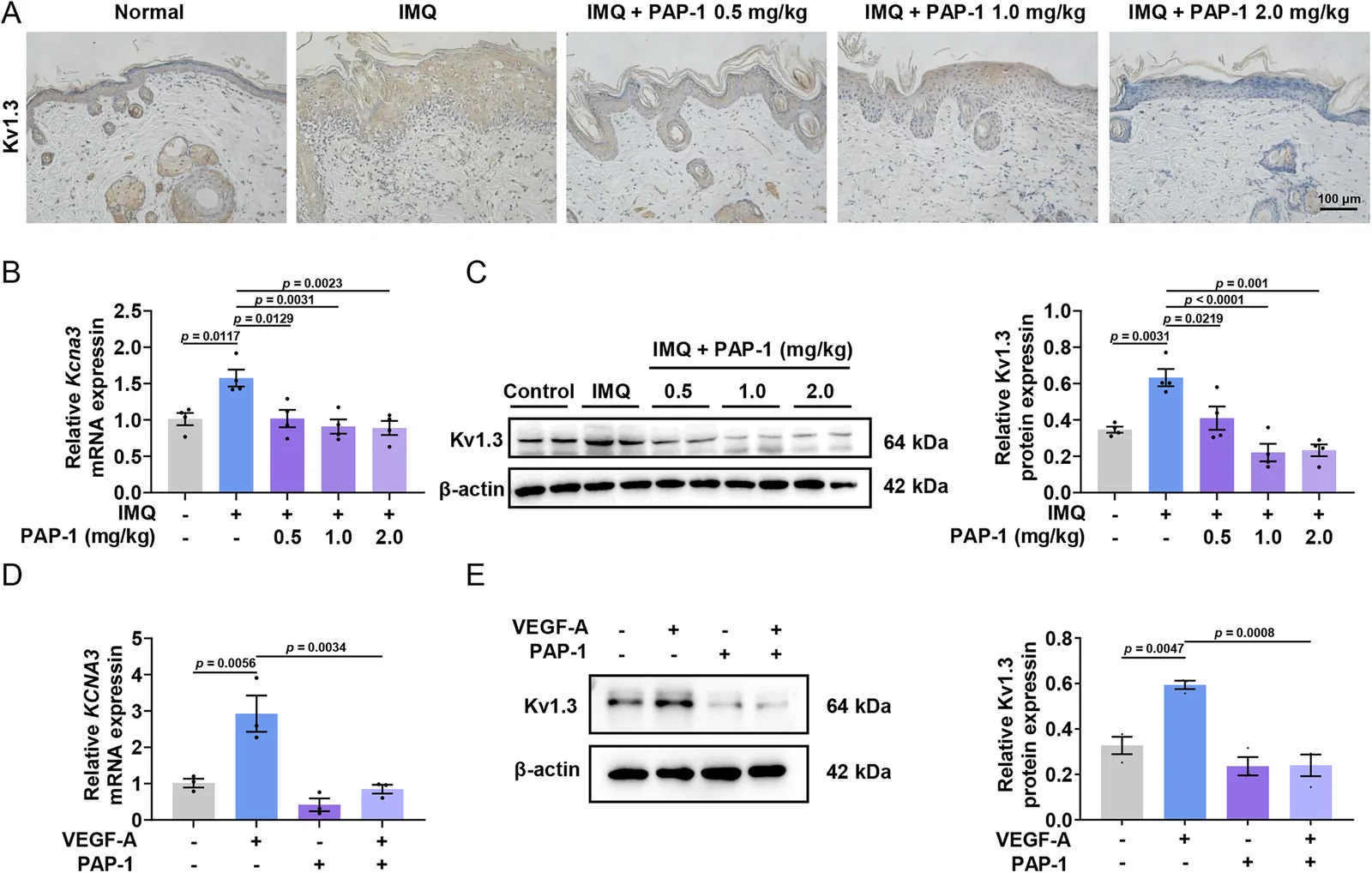
PAP-1 inhibits Kv1.3 expression in skin of IMQ-induced male rats and VEGF-A-induced HUVECs. **A** Immunohistochemical analysis of Kv1.3 expression in the affected dorsal skin of IMQ-induced psoriasis-like male rats (*n* = 4). Scale bar = 100 μm. **B** *Kcna3* mRNA expression in the affected dorsal skin of IMQ-induced psoriasis-like male rats measured by RT-PCR (*n* = 4). **C** Kv1.3 protein expression in the affected dorsal skin of IMQ-induced psoriasis-like male rats measured by Western blotting (*n* = 4). **D**, **E** HUVECs were treated with 10 nM PAP-1 in the presence of VEGF-A (10 ng/mL) for 48 h, followed by measurement of *KCNA3* mRNA and Kv1.3 protein expression using RT-PCR and Western blotting, respectively. *p* < 0.05 was considered statistically significant.

We investigated the effect of PAP-1 on Kv1.3 expression in the affected dorsal skin of IMQ-induced psoriasis-like male rats. Immunohistochemical analysis revealed increased Kv1.3 expression in the affected dorsal skin of IMQ-induced male rats compared to the normal group, whereas preventive treatment with 0.5, 1.0, and 2.0 mg/kg/d PAP-1 reduced Kv1.3 expression (Fig. 6A). Furthermore, *Kcna3* mRNA and Kv1.3 protein expression levels were significantly elevated in the affected dorsal skin of IMQ-induced male rats compared to the normal group, as assessed by RT-PCR and Western blotting. Preventive treatment with 0.5, 1.0, and 2.0 mg/kg/d PAP-1 markedly decreased *Kcna3* mRNA and Kv1.3 protein expression levels in the affected dorsal skin of IMQ-induced male rats (Fig. 6B, C). Additionally, we found that 10 ng/mL VEGF-A significantly increased *KCNA3* mRNA and Kv1.3 protein expression in HUVECs, while treatment with 10 nM PAP-1 significantly decreased *KCNA3* mRNA and Kv1.3 protein expression compared to the VEGF-A group (Fig. 6D, E). These findings confirm that PAP-1 inhibits Kv1.3 expression in skin of IMQ-induced male rats and VEGF-A-induced HUVECs, suggesting that PAP-1 inhibits angiogenesis and alleviates IMQ-induced psoriasis-like dermatitis by inhibiting the Kv1.3 channel.

### Structure of the Kv1.3/PAP-1 complex

**Fig. 7:**
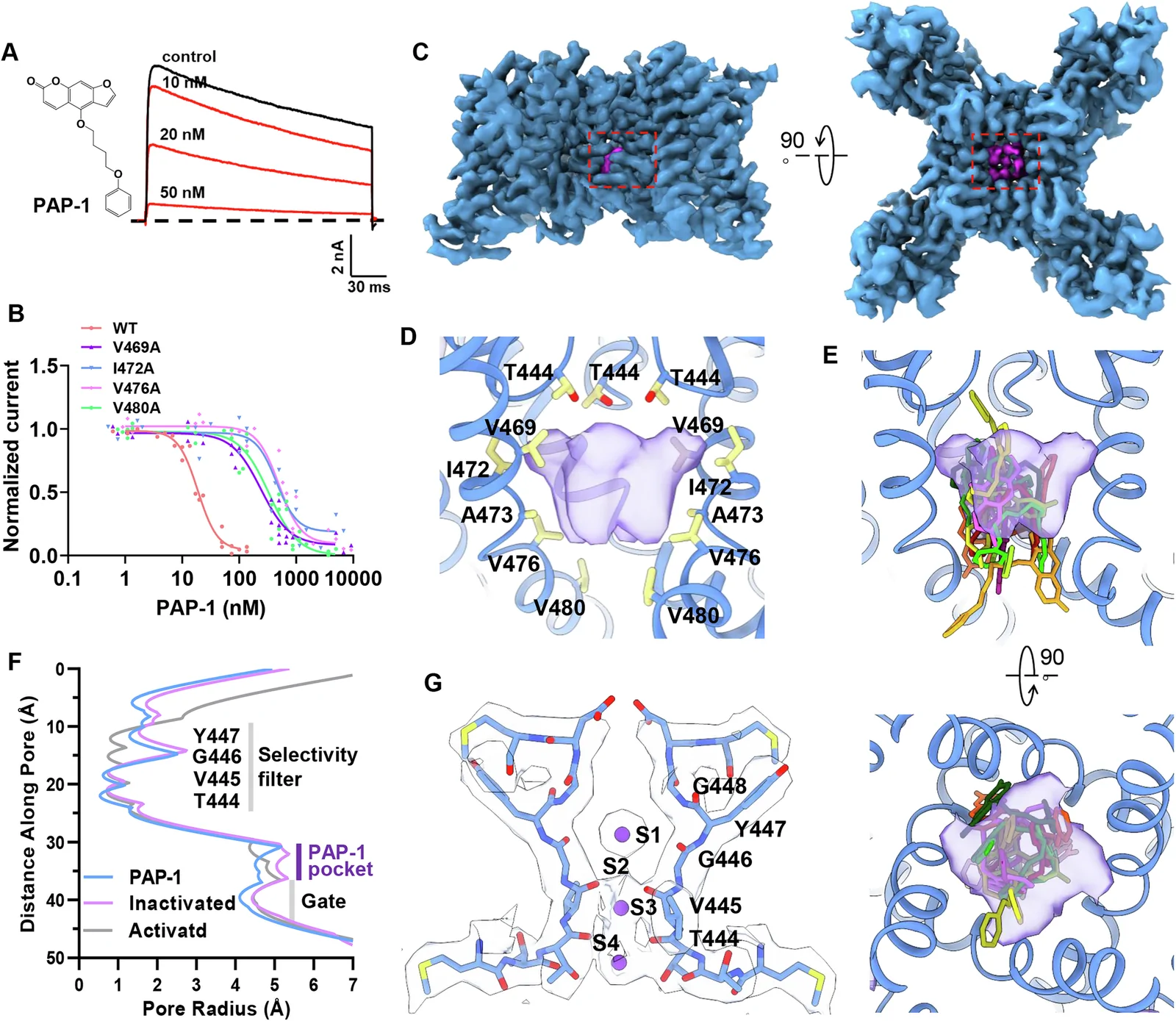
Whole-cell patch clamp electrophysiology and cryo-EM structure determination of the Kv1.3/PAP-1 complex. **A** Representative whole-cell recording traces from CHO cells expressing Kv1.3, showing inhibition of Kv1.3 current by increasing concentrations of PAP-1. **B** Concentration-response curve for PAP-1 on wild-type (WT) and mutant Kv1.3 channels. Each concentration was tested at least three times. The obtained IC_50_ values were as follows: WT, 18.8 ± 0.9 nM; V469A, 240.5 ± 41.1 nM; I472A, 452.0 ± 90.2 nM; V476A, 501.2 ± 65.4 nM; V480A, 334.5 ± 47.0 nM. **C** Cryo-EM map of the Kv1.3TMD/PAP-1 complex viewed from the membrane plane (top) and intracellular side (bottom). The density corresponding to the PAP-1 molecule is displayed in magenta. **D** Molecular details of the PAP-1-binding pocket viewed parallel to the membrane plane. The potential density corresponding to PAP-1 is shown as semi-transparent. For clarity, only three subunits are displayed. **E** Predicted PAP-1 binding conformations from docking and subsequent MD simulations are overlaid and viewed from the membrane plane (top) and extracellular side (bottom). **F** Plots of the pore radius for Kv1.3 channels (inactivated: PDB 7SSY; activated: PDB 7EJ1) along the pore axis, calculated using the HOLE program. **G** The selectivity filter of Kv1.3 bound to PAP-1 and the potassium ions are displayed with their corresponding densities contoured at 4σ. Potassium ions (purple spheres) are observed at sites S1, S3, and S4, but are missing at site S2.

We assessed the impact of PAP-1 on Kv1.3 currents using patch-clamp techniques. Whole-cell currents recorded from CHO cells expressing human Kv1.3 showed inhibition of Kv1.3 currents by increasing concentrations of PAP-1 (Fig. 7A). PAP-1 blocked Kv1.3 with an IC_50_ value of 18.8 nM (Fig. 7B).

To gain insights into the inhibition mechanism of PAP-1 on Kv1.3, we determined the cryo-EM structure of Kv1.3 in complex with PAP-1 at an overall resolution of 2.67 Å. Since the intracellular domain of Kv1.3 is not involved in PAP-1 binding, it was masked out during refinement to enhance the resolution of the transmembrane domain (TMD). The final reconstruction used for structural analysis therefore comprises only the TMD with an overall resolution of 2.76 Å (Supplementary Fig. 3 and Table 1). Within the central cavity of the channel pore, substantial additional density is present that most likely corresponds to the PAP-1 molecule (Fig. 7C). The PAP-1 binding pocket lies beneath the selective filter (TVGYG) and is lined by the hydrophobic residues V469, I472, A473, V476, and V480 on the Kv1.3 S6 helix, consistent with the overall hydrophobicity of the compound (Fig. 7D). However, the density for PAP-1 is poorly defined, probably because its inherent asymmetry conflicts with the fourfold symmetry of the Kv1.3 channel, creating a symmetry mismatch. PAP-1 can occupy any of the four structurally equivalent sites within the tetramer, and because its density constitutes only an extremely small fraction of the total complex and the signal-to-noise ratio for individual particles is low, it is not feasible to classify and obtain a PAP-1 structure in a single orientation. To further characterize the interaction, we also evaluated PAP-1 against several Kv1.3 mutants (Supplementary Fig. 4). The resulting IC₅₀ values were as follows: V469A, 240.5 ± 41.1 nM; I472A, 452.0 ± 90.2 nM; V476A, 501.2 ± 65.4 nM; V480A, 334.5 ± 47.0 nM (Fig. 7B). All mutations significantly reduced the inhibitory potency of PAP-1 against Kv1.3, with IC_50_ values increasing by 13- to 27- fold, strongly supporting the critical role of these residues in PAP1 binding.

**Table 1:** Statistics of cryo-EM data collection, 3D reconstruction, model refinement and validation

Data collection and processing	
Magnification	81,000
Voltage (kV)	300
Electron exposure (e^–^/Å^2^)	50
Defocus range (μm)	–1.2 to –2.4
Pixel size (Å)	1.07
Symmetry imposed	C4
Initial particle images (no.)	3,449,880
Final particle images (no.)	378,185
Map resolution (Å)	2.76
FSC threshold	0.143
**Refinement**	
Model resolution (Å)	3.02
FSC threshold	0.5
Map sharpening *B* factor (Å^2^)	–146.85
Model composition	
Non-hydrogen atoms	7653
Protein residues	975
Ligands	3 (K^+^)
*B* factors (Å^2^)	
Protein	112.16
Ligand	30.00 (K^+^)
R.m.s. deviations	
Bond lengths (Å)	0.002
Bond angles (°)	0.505
Validation	
MolProbity score	1.47
Clashscore	6.60
Poor rotamers (%)	0
Ramachandran plot	
Favored (%)	97.48
Allowed (%)	2.52
Disallowed (%)	0

These technical challenges preclude the construction of a PAP-1 model from the observed density. Consequently, we employed molecular docking and molecular dynamics (MD) simulations to model the binding mode of PAP-1 to Kv1.3 based on the cryo-EM density (Fig. 7E and Supplementary Fig. 5). The top 30 docking-predicted binding conformations of PAP-1 were classified into four classes (Supplementary Fig. 5A). Each class was subjected to subsequent MD simulations for three parallel runs (Supplementary Fig. 5B and C). The results showed that although PAP-1 remained in the central cavity beneath the selectivity filter, it displayed various conformations relative to the channel. Generally, the psoralen headgroup of PAP-1 inserts perpendicularly to the membrane plane, while the flexible phenyl tail was not in a fixed conformation and varied significantly (Fig. 7E).

The voltage-sensing domain (VSD) of Kv1.3 is in a depolarized conformation with the inner gate of the S6 helix in an open state (Fig. 7F and Supplementary Fig. 6). The selectivity filter is expanded at the top (Fig. 7F, G). In the density map, potassium ion density is observed at the S1, S3, and S4 coordination sites. Specifically, K^+^ ions at sites S3 and S4 are clearly seen to coordinate with backbone carbonyl oxygens. However, the K^+^ ion at site S1 does not appear to coordinate with the residues of the selectivity filter (Fig. 7G). To further evaluate the conformational state of the channel, we compared the Kv1.3/PAP-1 structure with representative Kv channel structures, including the conducting-like Kv1.2–2.1 paddle chimera structure (PDB 2R9R), and the dilated Kv1.3-D2 inactivated conformation (PDB 7SSY) (Supplementary Fig. 7). In theKv1.2-2.1 paddle chimera structure, the TVGYG selectivity filter adopts a canonical conductive geometry, with the backbone carbonyl oxygens pointing toward the pore axis, allowing each site to coordinate a potassium ion. By contrast, the selectivity filter of the Kv1.3/PAP-1 complex is widened at the extracellular side and shows altered potassium ion coordination. This architecture more closely resembles the dilated Kv1.3-D2 inactivated conformation. The conformation of the selectivity filter suggests that the structure is trapped in an inactivated state^8,13,14^ (Fig. 7F). This observation is consistent with previous studies indicating that PAP-1 preferentially binds to Kv1.3 in its inactivated state^10^.

## Discussion

Psoriasis is a chronic, inflammatory multisystem disease affecting approximately 2–4% of the global population^15^. Psoriasis is a lifelong disorder that all psoriatic patches and plaques decrease patients’ quality of life^16^. Despite advancements in targeted therapies, psoriasis remains incurable. In this study, we demonstrate that intraperitoneal injection of PAP‑1 significantly alleviates psoriasis‑like severity in IMQ‑induced male rats through integrated anti‑angiogenic and anti‑inflammatory mechanisms. We observed direct anti‑angiogenic effects of PAP‑1 in VEGF‑A‑stimulated HUVECs. Notably, PAP‑1 not only blocked Kv1.3 currents but also downregulated Kv1.3 expression in both the skin of IMQ‑induced male rats and VEGF‑A‑induced HUVECs. Structural analysis further revealed that PAP‑1 binds within the central cavity of Kv1.3, beneath the selectivity filter. Our study underscores the potential of PAP-1 for treating psoriasis and associated dermal inflammation and angiogenesis, as well as the mechanism of action of PAP-1 on Kv1.3.

IMQ is commonly used in mouse and rat models to simulate human psoriasis^17,18^. As a TLR7/8 agonist and potent immune activator, IMQ can induce and exacerbate psoriasis when topically applied to mouse and rat skin^19^. Given the limited data on sex differences, we selected male rats to control for variables, since hormonal fluctuations in females may affect immune responses. In the IMQ-induced male rat model, psoriasis-like skin conditions manifest, characterized by increased skin thickness and elevated severity scores for erythema and scaling. Preventive and therapeutic PAP-1 treatment ameliorated phenotypic and histological changes induced by IMQ, demonstrating its effective attenuation of psoriasis. Ki67 serves as a marker of cell proliferation, and its aberrant protein expression is notably increased in keratinocytes during psoriasis-like dermatitis^20^. Our findings further revealed that preventive and therapeutic PAP-1 treatment significantly reduced Ki67 expression in the skin of IMQ-induced psoriasis-like male rats, indicating its inhibition of keratinocyte proliferation in psoriatic conditions. Importantly, the reduction in CD3^+^T‑bet^+^ cell infiltration observed with both preventive and therapeutic PAP‑1 treatment indicates sustained suppression of pathogenic Th1‑type inflammation-a central driver of psoriatic pathogenesis. Previous studies have reported that PAP‑1 lacks cytotoxic and phototoxic effects, and only influences cytochrome P450‑dependent enzymes at micromolar concentrations, demonstrating a favorable safety profile^10^. Our safety assessments confirmed that PAP‑1, administered preventively or therapeutically, did not cause significant organ damage, weight loss, or renal impairment over 7 days. Notably, PAP‑1 treatment also reduced IMQ‑induced elevations in liver enzymes, further supporting its favorable safety profile. Moreover, according to the results of Non-invasive MRI, we found that in the process of psoriasis, the structural characteristics of the spleen changed obviously, and the thickness of the skin in the shaved area was also obviously thickened and the degree of wrinkling was intensified, and the preventive PAP-1 treatment can have a certain control effect on the volume and size of the spleen. It can be seen from the image of signal distribution that PAP-1 obviously increased the water content of the spleen and skin, which indicated that preventive PAP-1 treatment had a certain control effect on the course of psoriasis. Although we should further expand the number of MRI studies in animals to get more accurate results, these findings initially revealed the role of PAP-1 in alleviating psoriasis-like dermatitis in IMQ-induced male rats.

The spleen, as the largest secondary lymphoid organ in the immune system, reflects systemic inflammatory response through its spleen index^21^. Preventive and therapeutic PAP-1 treatment reduced the IMQ-induced spleen index, highlighting its anti-inflammatory effects. Psoriasis is characterized by various pro-inflammatory cytokines that modulate inflammatory responses^22,23^. In the skin, IL-17A acts on cellular targets such as keratinocytes, neutrophils, and endothelial cells, stimulating the production of chemokines, pro-inflammatory, and proliferative cytokines, thereby promoting tissue inflammation^24^. Studies have shown that IL-33 exacerbates disease severity in psoriasis-like mouse models, and anti-IL-33 antibodies have been demonstrated to ameliorate erythema, scaling, epidermal thickness, and spleen index in IMQ-induced psoriatic dermatitis in mice^25^. Patients with psoriasis exhibit elevated plasma IL-6 levels compared to healthy controls, with IL-6 contributing to disease severity^26,27^. A phase Ib trial demonstrated that the Kv1.3 channel inhibitor dalatazide improved psoriatic skin lesions by modulating T cell surface markers, inhibiting activation markers, and reducing pro-inflammatory mediators in the^28^. Recent studies have also shown that Kv1.3 blockade attenuates expression of pro-inflammatory cytokines in angiotensin II-induced hypertension in mice^29^. Our present results indicate that treatment with the Kv1.3 blocker PAP-1 significantly decreased levels of pro-inflammatory cytokines, including IL-17A, IL-33, and IL-6, in both serum and skin of IMQ-induced male rats.

In the development of psoriasis, vascular changes precede epidermal proliferation. VEGF-A, a crucial angiogenic mediator, is upregulated in both plaques and plasma of psoriasis patients^30^. Studies have shown that treatment with anti-VEGF antibodies led to overall improvement in psoriatic lesions, marked reduction of inflammatory cells in the dermis, and normalization of epidermal differentiation in mice displaying disease symptoms^31^. Another study highlighted the potent angiogenic activity within psoriatic lesions, evidenced by increased levels of VEGF-A and CD31 in the skin^32^. Our results indicate that both preventive and therapeutic administration of PAP‑1 reduce VEGF‑A and CD31 levels in the skin of IMQ‑induced psoriasis‑like male rats. Dermal microvascular endothelial cells (DMECs), though ideal for studying vascular lesions in skin diseases, are challenging to culture due to contamination risks from mesenchymal stem cells, fibroblasts, and other stromal cells, limiting their application^33^. However, primary DMECs are difficult to culture and often contaminated by mesenchymal stem cells, fibroblasts, and other stromal cells, so its application is limited^34^. In our study, we utilized HUVECs as a widely accepted substitute for DMECs in psoriasis research. Previous research has shown that PAP-1 treatment reduces smooth muscle cell proliferation in arteriogenesis^35^. It has been found that PAP-1 decreased smooth muscle cells proliferation, migration, and in vivo intimal hyperplasia formation^36^. Our results further demonstrate that PAP-1 inhibits VEGF-A-induced tube formation of HUVECs by reducing cell proliferation, migration, and invasion.

PAP-1 was identified as binding at the center of the inactivated channel pore beneath the selective filter and above the gate, in a region lined by a number of hydrophobic residues on the Kv1.3 S6 helix. Consistent with its high-affinity binding observed structurally, our electrophysiological recordings confirmed that PAP-1 is a potent blocker of Kv1.3, with an IC₅₀ of 18.7 nM in our experimental system. This value is consistent with the high-potency, low-nanomolar range (e.g., 2 nM) reported in prior studies^37^, with the observed difference likely attributable to variations in cell type or recording conditions, collectively supporting the robust pharmacological profile of PAP-1 across independent assays. To further characterize the interaction, we evaluated PAP-1 against several Kv1.3 mutants. All four mutations (V469A, I472A, V476A, V480A) substantially attenuated PAP-1 inhibition, providing evidence that these residues are important for PAP-1 binding. In a previous study, researchers proposed that two (or possibly up to four) PAP-1 molecules coordinate a K^+^ ion in the central cavity of Kv1.3 beneath the selectivity filter, with their long flexible phenoxyalkoxy side chains protruding into the intrasubunit interfaces between helices S5 and S6^37^. Another study on Psora-4, a structural template for PAP-1. reported that in addition to binding the central pore cavity, this compound also occupies a secondary, less conserved site in Kv1.5 side pockets formed by S5, S6, the S4-S5 linker, and the pore helix^38^. As mentioned above, our cryo-EM density for PAP-1 is poorly defined due to symmetry mismatch: PAP-1 can occupy any of the four equivalent sites within the tetramer, and its density constitutes only a tiny fraction of the complex, precluding determination of its orientation. We observed density consistent with at least one PAP-1 molecule below the selectivity filter, but no additional discrete densities that could be confidently assigned to a second molecule. Importantly, PAP-1 binding does not induce a major global rearrangement of the channel architecture. Instead, PAP-1 occupies the central cavity of the pore, supporting steric occlusion of the ion-conduction pathway as the primary mechanism of inhibition. We therefore interpret that PAP-1 preferentially binds to, and likely stabilizes, a pre-existing inactivated-like selectivity filter conformation over the active state. While our structural data support a primary high-affinity binding site directly occluding the pore, more complex pharmacology remains to be fully captured by our current model, warranting future investigation.

In summary, our integrated data demonstrate that the Kv1.3 blocker PAP‑1 alleviates IMQ‑induced psoriasis‑like dermatitis in male rats by suppressing keratinocyte proliferation, inflammatory responses, and angiogenesis through dual anti‑inflammatory and anti‑angiogenic mechanisms. While the intraperitoneal route used here is more invasive than topical delivery, the doses were well tolerated with no acute toxicity or significant histopathology in major organs. Future studies should include long‑term safety assessments and explore optimized formulations for clinical translation. These findings provide a strong rationale for advancing PAP‑1 as a potential psoriasis therapeutic and underscore the broader relevance of Kv1.3 channel modulation in treating this disease.

## Materials and methods

### IMQ-induced psoriasis-like rat model and PASI assessment

Male Sprague-Dawley rats (*n* = 48, weighing 160–200 g, aged 6–8 weeks) were obtained from Hangzhou Ziyuan Experimental Animal Technology Co., Ltd (Zhejiang, China). All animal procedures were approved by the Animal Ethics Committee of the Hefei Institutes of Physical Science, Chinese Academy of Sciences (DWLL(E)-2024-04). We have complied with all relevant ethical regulations for animal use. The male rats were maintained under pathogen-free conditions, and all experiments were conducted according to Animal Care and Use Committee guidelines. We first established the preventive and concurrent intervention model. After a one-week adaptation period, the male rats (*n* = 6 per group) were randomly divided into five groups using a computer‑generated random sequence: a normal group, an IMQ group, and three PAP‑1 (HY-10015, MedChemExpress, New Jersey, USA) preventive administration groups (0.5, 1.0, and 2.0 mg/kg). At the start of the experiment, the hair on the back (2.5 cm × 2 cm area) was shaved. The normal group received a daily application of control cream (Vaseline cream, Unilever), whereas the IMQ group and the PAP‑1 preventive groups received a daily topical dose of 125 mg of 5% IMQ cream (MED-SHINE, Sichuan, China) on the shaved area^39^. Concurrently, the PAP‑1 preventive groups were injected intraperitoneally with PAP‑1 at the respective doses once daily, while the IMQ group received an equal volume of vehicle via the same route. After six consecutive days of intervention, photographs of the back skin were taken and tissue samples were collected for analysis. To evaluate the therapeutic effect of PAP‑1, an IMQ‑induced skin lesion model was first established. Male Sprague‑Dawley male rats (*n* = 12) received a daily topical application of 5% IMQ cream on the shaved dorsal area for six consecutive days. After successful modeling, the male rats were randomly divided into three groups (*n* = 6 per group): an IMQ model group (no treatment), a PAP‑1 treatment group, and a normal control group (without modeling). 6 male rats per group were used, consistent with standard practice in preclinical psoriasis research. This sample size provides adequate statistical power for key endpoints and has been approved by the animal ethics committee. The PAP‑1 treatment group was administered 2.0 mg/kg PAP‑1 once daily by intraperitoneal injection for seven consecutive days, while the IMQ model group received an equal volume of vehicle during the same period. The degree of erythema, thickness, and scaling on the affected dorsal skin surface was evaluated to determine the PASI scores. The PASI was assessed on the affected dorsal skin based on erythema, thickness, and scaling. Scoring was performed by investigators blinded to group assignment, with each parameter rated 0–4 (0 = none, 1 = slight, 2 = moderate, 3  = marked, 4 = very marked). The cumulative score (0–12) represented the sum of the three subscores. No animals or data were excluded; all subjects completed the study and were included in the analysis. At the endpoint, animals were anesthetized with sodium pentobarbital (45 mg/kg, i.p.) and then euthanized by cervical dislocation.

### H&E staining

Samples, including skin, heart, liver, spleen, lung, and kidney, were harvested and fixed in 4% paraformaldehyde solution before being embedded in paraffin. The paraffin-embedded sections were used for H&E staining and observed under a microscope (AX10, Carl Zeiss, Germany). The epidermal thickness of the shaved back skin of the male rats was measured using ImageJ software (National Institutes of Health, USA).

### Immunofluorescence and immunohistochemistry

Paraffin-embedded sections of affected dorsal skin (*n* = 4 per group) were processed for immunofluorescence and immunohistochemical analysis. For immunofluorescence, sections were permeabilized with 0.5% Triton X-100 (P0096, Beyotime, Haimen, China) for 30 min, followed by immersion in EDTA antigen retrieval buffer (P0098‌, Beyotime, Haimen, China), and blocking with 5% BSA (4240GR250, Biofroxx, Einhausen, Germany) for 1 h at room temperature. Subsequently, sections were incubated overnight at 4 °C with a Ki67 polyclonal antibody (1: 100; 28074-1-AP, Proteintech, Wuhan, China). For double immunofluorescence, sections were incubated overnight at 4 °C with Ki67 polyclonal antibody (1: 100; 28074-1-AP), or a combination of anti-CD3 mouse antibody (1: 500; GB12014-50, Servicebio, Wuhan, China) and anti-T-bet rabbit polyclonal antibody (1: 500; ab307193, abcam, Cambridge, UK). Following primary antibody incubation, sections were treated with Alexa Fluor® 488-conjugated goat anti-rabbit IgG (H + L) (1: 200; ZF-0511, ZSGB-BIO, Beijing, China) and Alexa Fluor® 594-conjugated goat anti-mouse IgG (H + L) (1: 200; ZF-0513, ZSGB-BIO, Beijing, China). Nuclei were counterstained with DAPI (Beyotime, Haimen, China). Following routine immunofluorescence procedures, the samples were observed under a fluorescence microscope (AX10, Carl Zeiss, Germany). For immunohistochemistry, sections were incubated overnight at 4 °C with primary antibodies against Kv1.3 (1: 100; 14079-1-AP, Proteintech, Wuhan, China), VEGF-A (1: 100; 19003-1-AP, Proteintech, Wuhan, China), or CD31 (1:100; GB12063-50, Servicebio, Wuhan, China). Subsequently, sections were incubated with a horseradish peroxidase-labeled secondary antibody at 37  °C for 30 min, followed by hematoxylin counterstaining for 60 s. Finally, the samples were examined under a microscope.

### MRI

MRI studies were performed on different groups of experimental male rats. In order to collect high-resolution MRI data, MRI system (Bruker Bios pin MRI, Ettlingen, Germany) was used. The T1-weighted image (T1WI) and T2-weighted image (T2WI) sequences of the spleen region and the skin area of the shaved area of each rat were collected at 9.4 T (Agilent Technologies Inc., Santa Clara, CA). The parameters of the T1WI sequence in the skin area of the shaved area are as follows: TE/TR = 3/500 ms, Averages = 5, Repetitions = 1, Flip Angle = 50°, Thickness = 1 mm, slices = 25, Fov =  40 × 70 mm, Image Size = 334 × 438, Bandwidth = 39682.5 Hz, Scan time = 13 m 40 s. The parameters of the T_2_WI sequence in the skin area of the shaved area are as follows: TE/TR = 27.1/4000 ms, Averages = 5, Repetitions = 1, Rare Factor = 8, Thickness = 1 mm, slices = 25, Fov = 40 × 70 mm, Image Size = 334 × 438, Bandwidth = 50,000 Hz, Scan time = 18 m. The parameters of the T_1_WI sequence in the spleen region are as follows:TE/TR = 3/500 ms, Averages = 5, Repetitions = 1, Flip Angle = 50 °, Thickness = 1 mm,slices = 25, Fov = 40 × 70 mm, Image Size = 200 × 350, Bandwidth = 39682.5 Hz, Scan time = 10 m 55 s. The parameters of the T_2_WI sequence in the spleen region are as follows: TE/TR = 20/4000 ms, Averages = 5, Repetitions = 1, Rare Factor = 8, Thickness = 1 mm, slices = 25, Fov = 40 × 70 mm, Image Size = 200 × 350, Bandwidth = 50,000 Hz, Scan time = 14 m 20 s. All sequences have a fat pressing option added.

### Cytokine analysis by ELISA

Blood samples were collected and serum was separated by centrifugation at 3000 rpm for 15 min. The concentrations of IL-17A, IL-33, IL-6, and VEGF-A in the rat serum (*n* = 4 per group) were measured using commercial ELISA kits (RX302871R, RX302873R, RX302872, and RX202522R; Ruixin Biotech, Quanzhou, China) following the manufacturer’s instructions. Absorbance was measured at a wavelength of 450 nm using a microplate reader (Agilent Technologies, Germany).

### RNA sequencing

Total RNA was extracted from the affected dorsal skins (*n* = 3 per group) using the Trizol Reagent (15596-026, Invitrogen Life Technologies) following the manufacturer’s protocol. RNA purity and quantification were assessed using the NanoDrop spectrophotometer (Thermo Scientific, USA). Starting with 3 μg of RNA, mRNA was enriched using poly-T magnetic beads, fragmented, and reverse-transcribed into double-stranded cDNA, followed by end repair, A-tailing, adapter ligation, size selection (400–500 bp), 15-cycle PCR amplification, purification, and quantification, before sequencing on the NovaSeq Xplus platform. Transcriptome sequencing and analysis were conducted by Bioprofile (Shanghai, China). Sequencing images are converted to raw FASTQ data, which is then filtered using Cutadapt (v1.15) to remove adapters and low-quality reads, yielding clean data for further analysis. Reference genome and annotation files were downloaded from a genome website, and clean reads were mapped to the reference genome using HISAT2 v2.0.5; HTSeq (0.9.1) was then used to count reads per gene, FPKM for normalization, and DESeq2 (1.30.0) for differential expression analysis (|log_2_FoldChange | >1 and *p* < 0.05), followed by bidirectional clustering using the R package pheatmap (1.0.8) with Euclidean distance and complete linkage. For functional enrichment analysis, GO and KEGG pathway analyses of DEGs were performed using R based on the hypergeometric distribution.

### Cell culture and cell viability assay

HUVECs were obtained from the American Type Culture Collection (CRL-1730, Manassas, USA). The cells were cultured in DMEM/F-12 medium (11320-033, Gibco) supplemented with 10% FBS (10099-141 C, Gibco) and 100 IU/mL penicillin-streptomycin (C0222, Beyotime, China) in a humidified incubator at 37 °C with 5% CO_2_. Cell viability was assessed using a CCK-8 assay. HUVECs were seeded at a density of 1 ×  10^4^ cells per well in 96-well plates with complete medium and treated with various concentrations of PAP-1 (0, 2.5, 5, 10, 20, 40, and 80 nM) in the presence or absence of 10 ng/mL VEGF-A (HY-P7110A, MedChemExpress) for 48 h. After incubation, 10 μL of CCK-8 solution was added to each well, and absorbance was measured at 450 nm using a microplate reader.

### Tube formation assay

HUVECs were seeded into a 12-well plate and allowed to grow to nearly 70% confluence. They were then exposed to VEGF-A (10 ng/mL) in combination with PAP-1 (10 nM) for 48 h. Subsequently, HUVECs were seeded at a density of 2 × 10^4^ cells per well onto a layer of Matrigel in 96-well plates and incubated for 4 h. The tube formation was assessed by analyzing vessel area, total number of junctions, and total vessel length using AngioTool64 version 0.6a (Maryland, USA). Images were captured using a microscope.

### Wound closure assay

For the scratch assay, HUVECs were plated into a 12-well plate and grown to over 90% confluence. A scratch was made in the cell monolayer using a sterile plastic pipette tip, and the culture medium was replaced with serum-free DMEM/F-12 medium. The change in scratch width was monitored and recorded at 0 h, 24 h, and 48 h under a fluorescence microscope. The wound closure rate was calculated as [(width at 0 h) - (width at 24 or 48 h)] / (width at 0 h).

### Cell migration and invasion

Transwell chambers (3422, Corning, New York, USA) were employed to assess cell migration and invasion. HUVECs were detached using trypsin and suspended in DMEM/F12 medium supplemented with 5% FBS to a final concentration of 2 × 10^4^ cells/mL. Subsequently, 200 μL of the cell suspension was added to the upper chamber, while 600 μL of complete medium was added to the lower wells. For the invasion assay, similar procedures were followed using inserts coated with Matrigel (354234, Corning, New York, USA). After incubation for 48 h, non-migratory cells in the upper chamber were removed with a cotton swab, and cells adhering to the lower surface were fixed with methanol for 20 min and stained with crystal violet for 60 min. Stained cells were observed and photographed under a microscope.

### RNA extract and real-time polymerase chain reaction

Total RNA was isolated from the affected dorsal skins (*n* = 4 per group) using RNAex Pro RNA extraction reagent (AG21101‌, Accurate Biology, Hunan, China) following the manufacturer’s instructions. RNA concentration was quantified and reverse-transcribed to cDNA using the 5 × Master Mix (AM1005, Accurate Biology, Hunan, China). RT-PCR was performed with the SYBR Green Pro Taq HS Premixed qPCR Kit (AG11701‌‌, Accurate Biology, Hunan, China). Gene expression levels were normalized to GAPDH expression. Relative gene expression was determined using the comparative Ct method (2^−ΔΔCT^). Specific primer sequences are listed in Supplementary Table 1.

### Western blotting

The affected dorsal skins (*n* = 4 per group) and HUVECs were homogenized in RIPA lysis buffer (P0013B, Beyotime, Haimen, China) supplemented with protease inhibitor cocktail (ST506, Beyotime, Haimen, China). Total protein was extracted by centrifugation at 12,000 rpm for 15 min. Protein samples were separated on a 10% SDS-PAGE gel and transferred onto polyvinylidene difluoride membranes. The membranes were blocked with TBST containing 5% skim milk for 2 h at room temperature, followed by overnight incubation at 4  °C with Kv1.3 polyclonal antibody (1: 1000; 14079-1-AP, Proteintech, Wuhan, China) or β-actin monoclonal antibody (1: 2000; YM3028, ImmunoWay, Texas, USA). Subsequently, the membranes were incubated with secondary goat anti-rabbit (1: 5000; ZB-2301, ZSGB-BIO, Beijing, China) or anti-mouse (1: 5000; ZB-2305, ZSGB-BIO, Beijing, China) IgG antibodies for 2 h at room temperature. The blots were visualized using enhanced chemiluminescent substrate and quantified using ImageJ software (National Institutes of Health, USA).

### Electrophysiology studies

CHO cells were obtained from the American Type Culture Collection (CHO-K1, Manassas, USA), and were cultured in DMEM/F12 medium (11320-033, Gibco) supplemented with 10% FBS (10099-141 C, Gibco), and 1% Penicillin-Streptomycin-Glutamine (10378016‌, Gibco) at 37  °C in a 5% CO_2_ incubator. The cDNA encoding human Kv1.3 was cloned into the pcDNA3.1/Zeo (+) vector. Site‑directed mutants (V469A, I472A, V476A, and V480A) were generated using circular plasmid PCR. Plasmids carrying wild‑type Kv1.3 or the respective mutant, together with an EGFP reporter, were transiently transfected into CHO cells using Lipofectamine 3000 (Invitrogen). After incubation for 5 h, the cells were transferred to poly-L-lysine-coated glass coverslips and cultured for another 24–48 h in fresh medium. The cells were then used for electrophysiological recording.

The effects of PAP-1 on Kv1.3 were evaluated by whole-cell patch clamp using an EPC10 patch clamp amplifier (HEKA Electronics). The electrodes were pulled from thick-walled borosilicate glass capillaries with filaments (World Precision Instruments, Inc.) on a four-stage puller (P-1000, Sutter Instruments) and had resistances of 3-5 MΩ. Currents for Kv1.3 were evoked by 200-ms depolarizing pulses from a holding potential of -80 mV to 40 mV, repeated at 30-s intervals. After three stable records of currents, PAP-1 was applied to the patched cell through a Y-tube perfusion system, and the degree of block was determined. The measured reduction in peak current for each concentration was used to generate a dose-response curve. The recording solution compositions are as follows: the external solution: 150 mM NaCl, 4 mM KCl, 2 mM CaCl_2_, 1 mM MgCl_2_, and 10 mM HEPES (pH 7.4, ~308 mOsm); the internal solution: 140 mM KCl, 10 mM NaCl, 5 mM EGTA, and 10 mM HEPES (pH 7.4, ~297 mOsm). All chemicals were obtained from Sigma.

### Protein expression and purification

The protein was prepared as described in our previous paper^3^. Specifically, the gene for human Kv1.3 (UniProtKB accession no. P22001), lacking the first 52 residues, and human Kvβ2.1 (UniProtKB accession no. Q13303) were cloned for coexpression into a pFastBac dual vector (Invitrogen) with a FLAG tag (DYKDDDDK) at the N-terminus of Kv1.3. The expression construct for Kv1.3–Kvβ2.1 was transformed into DH10Bac cells to produce bacmid. For large-scale expression, baculovirus, after two rounds of amplification, was used to infect Sf9 cells. Cells were infected at a density of 4 × 10^6^ cells per mL of culture and harvested 60 h post-infection. The cell pellet from 1 L of culture was resuspended in lysis buffer (20 mM HEPES, pH 7.5, 150 mM KCl, and 10% glycerol). The suspension was supplemented with 1.5% n-dodecyl-β-D-maltopyranoside (D310, DDM, Anatrace, USA), 0.3% cholesteryl hemisuccinate (C6512, CHS, Sigma-Aldrich, Darmstadt, Germany), and protease inhibitor cocktail. After extraction at 4  °C for 2 h, the insoluble fraction was removed by ultracentrifugation at 180,000 × *g* for 45 min at 4  °C. The supernatant was incubated with anti-FLAG G1 affinity gel (L00430, GenScript, Nanjing, China) for 1 h at 4  °C. The resin was then collected and washed with wash (W) buffer (20 mM HEPES, pH 7.5, 150 mM KCl, and 0.1% GDN). The protein was eluted with W buffer containing 200 μg/mL FLAG peptide. After elution, the protein was concentrated and further purified on a Superose 6 Increase column (Cytiva) equilibrated with 20 mM HEPES, pH 7.5, 150 mM KCl, and 0.005% GDN. Peak fractions were collected, and PAP-1 was added at a molar ratio of 10:1. The mixture was incubated for 30 min and then concentrated to ~7 mg/ml before cryo-EM sample verification.

### Cryo-EM sample preparation and data acquisition

Total 3 μL of protein sample was applied to freshly plasma-cleaned (H2/O2, 10 s) holey carbon grids (Quantifoil, R1.2/1.3, 300 mesh, Cu). The grids were blotted for 4 s with a blot force of 1 at 4  °C and 100% humidity using a Vitrobot Mark IV (Thermo Fisher Scientific) and then plunge-frozen into liquid ethane cooled by liquid nitrogen. The blotted grids were stored in liquid nitrogen until imaged.

For data collection, Cryo-EM imaging was performed on a Titan Krios microscope at 300 kV, equipped with a Gatan K3 Summit direct electron detector and a Gatan energy filter. Movies were recorded at a magnification of SA81,000×, yielding a pixel size of 1.07 Å at the University of Science and Technology of China. In total, 4637 movies were obtained at a total dose of 50 electron per Å2 over 32 frames. The defocus values ranged from –1.2 to –2.4 μm.

### Cryo-EM data processing

Dose-fractionated image stacks were subjected to beam-induced motion correction and dose-weighting using UCSF MotionCor2^40^. The processed images were then subjected to CryoSPARC^41^ for contrast transfer function (CTF) estimation using patch CTF estimation. Following CTF estimation, automated particle selection yielded 3,449,880 particles for further processing. The auto-picked particles were extracted and subjected to reference-free 2D classification. After two rounds of 2D classification to discard poorly defined particles, 487,171 particles from well-defined 2D averages were selected and combined for further ab-initio reconstruction and heterogeneous refinement. An ab initio 3D reconstruction from the 2D average particles was generated in cryoSPARC. The initial model was then used as a reference for 3D classification by heterogeneous refinement with imposing C4 symmetry. A selected class with continuous density for all transmembrane helices was used to perform further 3D classification by heterogeneous refinement. Particles in the well-defined class were subjected to subsequent homogeneous refinement, and non-uniform refinement yielded a reconstruction with an overall resolution of 2.67 Å. Local refinements were further performed on transmembrane domain (TMD), which further improved the resolution of the TMD to 2.76 Å.

### Model building, refinement and validation

The model of apo-Kv1.3 (PDB: 7EJ1) was fitted into the Kv1.3-PAP1 map using UCSF ChimeraX. Every residue was manually examined. The initial model was subjected to iterative manual rebuilding in Coot and real-space refinement in PHENIX. The final refinement statistics were validated using the “comprehensive validation (cryo-EM)” module in PHENIX. The S1-S2 linker and S3-S4 linker were not built due to the lack of corresponding densities.

### Molecular docking

To gain insight into the binding model of PAP-1 within the central cavity of Kv1.3, we performed molecular docking using AutoDock Vina 1.2.4^42,43^ based on the cryo-EM density. The ADFR software suite was used to prepare the PDBQT format of Kv1.3 and PAP-1. The docking grid was set according to density of PAP-1. Finally, the docking models were visualized using PyMOL, and the top 30 docking-predicted binding conformations of PAP-1 were classified into four classes based on their conformations.

### Molecular dynamics simulation

The models from each class obtained from docking were subjected to molecular dynamics simulations. Four simulation systems were constructed using the membrane builder tool of the CHARMM-GUI website (http://www.charmm-gui.org/)^44^. As detailed in Supplementary Table 2, each system contains the Kv1.3/PAP‑1 complex, a POPC lipid membrane, water molecules, and 150 mM NaCl, with a total of approximately 127,000 atoms. A minimum water layer of 15 Å was maintained above and below the lipid bilayer, resulting in a simulation box of 125 Å × 125 Å × 90 Å. The CHARMM36m force field was used for the protein, lipids, and ions^45^. Waters were modeled using the TIP3P model. All equilibration and production simulations were performed and analyzed using Amber20 and AmberTools21. The CHARMM-GUI setup was followed to perform initial energy minimization and equilibration steps. Subsequently, each system underwent three parallel 500 ns production runs. Our molecular dynamics simulations were designed solely to observe conformational changes of PAP‑1, rather than to predict binding modes or to sample rare events. The timescale of these fluctuations was sufficiently covered by our total simulation time; therefore, enhanced sampling methods were unnecessary.

### Statistics and reproducibility

The data were analyzed using GraphPad Prism 8 (GraphPad Software, La Jolla, CA, USA) and presented as mean ± SEM. All cell experiments were performed with at least three biological replicates. Animal data from each group (*n* = 6 or 4 male rats) were statistically analyzed. Differences between groups were assessed using a t-test or one-way analysis of variance (ANOVA). Statistical significance was defined as a *p* value < 0.05.

### Reporting summary

Further information on research design is available in the Nature Portfolio Reporting Summary linked to this article.

## Supplementary information

Supplementary Material

Description of Additional Supplementary Files

Supplementary Data 1

Supplementary Data 2

Supplementary Data 3

Reporting Summary

## Data Availability

The data are available within the article and its Supplementary Material and Data. The raw sequence data have been deposited in the Genome Sequence Archive (GSA) under accession number CRA044579. The cryo-EM density map of the Kv1.3/PAP‑1 complex has been deposited in the Electron Microscopy Data Bank (EMDB) under accession code EMD-67655. The corresponding atomic coordinates have been deposited in the Protein Data Bank (PDB) under accession code 21GK. Uncropped and unedited blot images are shown in Supplementary Fig. 8. Supplementary Data 1 contains the initial coordinates, the simulation input files, and a coordinate file of the final output of molecular dynamics simulations. Supplementary Data 2 provides the plasmid sequence and its corresponding map. Supplementary Data 3 contains the source data underlying graphs. All other data supporting the findings of this study are available from the corresponding author upon reasonable request.
