# Supplementary material for: The Kv1.3 blocker PAP‑1 alleviates psoriasis‑like dermatitis in rats via suppressing inflammation and angiogenesis
Source: Commun Biol. 2026 Jul 20;9:1497. doi: 10.1038/s42003-026-10718-7 (PMC13748653; doi:10.1038/s42003-026-10718-7)
Supplement: Supplementary file 1 [file 42003_2026_10718_MOESM1_ESM.pdf]

Supplementary Materials for

The Kv1.3 blocker PAP-1 alleviates psoriasis-like dermatitis in rats via suppressing inflammation and angiogenesis

Feng Yao [#], Yong Zhang [#], Yanmin Zheng [#], Renpeng Zhou, Tianqi Liu, Lulu Wang, Gengxin Wang, Qiuxia Yu, Demeng Sun, Sanling Liu*, Changlin Tian*, Wei Hu*

The File includes:

Supplementary Tables 1-2;

Supplementary Figures 1-8.

Supplementary Table 1. Primer sequences used in real-time PCR

Gene	Primer	Sequence (5'-3')
Gapdh (Rat)	F	GACATGCCGCCTGGAGAAAC
	R	AGCCCAGGATGCCCTTTAGT
Kcna3 (Rat)	F	CTTCGACGCCATCCTCTACT
	R	GTCCTCACGGAACTTCTCCA
Il17a (Rat)	F	AGTTCAGTGTGTCCAAACGC
	R	GAGTCCAGGGTGAAGTGGAA
Il33 (Rat)	F	CAGACATCTGGCTGAATGCC
	R	CTGATCTGGCGGAGAGACAT
Il6 (Rat)	F	CTCTCCGCAAGAGACTTCCA
	R	TGAAGTAGGGAAGGCAGTGG
GAPDH (Human)	F	GGAGCGAGATCCCTCCAAAAT
	R	GGCTGTTGTCATACTTCTCATGG
KCNA3 (Human)	F	AGTTCCGCGACGAGAAGGA
	R	CGAAGAAGGGATCGGAGAAGC

Supplementary Table 2. Molecular dynamics simulation system setup

**Model 1**	
Num of Atoms	126900
Crystlal Type	tetragonal
System Size	x = y = 125.07, z = 89.22
Num of Lipids	POPC, on top 160, on bottom164
Num of Water	22499
salt concentration	150 mM NaCl
**Model 2**	
Num of Atoms	126990
Crystlal Type	tetragonal
System Size	x = y = 125.04, z = 89.36
Num of Lipids	POPC, on top 160, on bottom164
Num of Water	22529
salt concentration	150 mM NaCl
**Model 3**	
Num of Atoms	127138
Crystlal Type	tetragonal
System Size	x = y = 125.15, z = 89.36
Num of Lipids	POPC, on top 160, on bottom165
Num of Water	22533
salt concentration	150 mM NaCl
**Model 4**	
Num of Atoms	126816
Crystlal Type	tetragonal
System Size	x = y = 125.06, z = 89.36
Num of Lipids	POPC, on top 160, on bottom164
Num of Water	22471
salt concentration	150 mM NaCl

Figures and Figure Legends

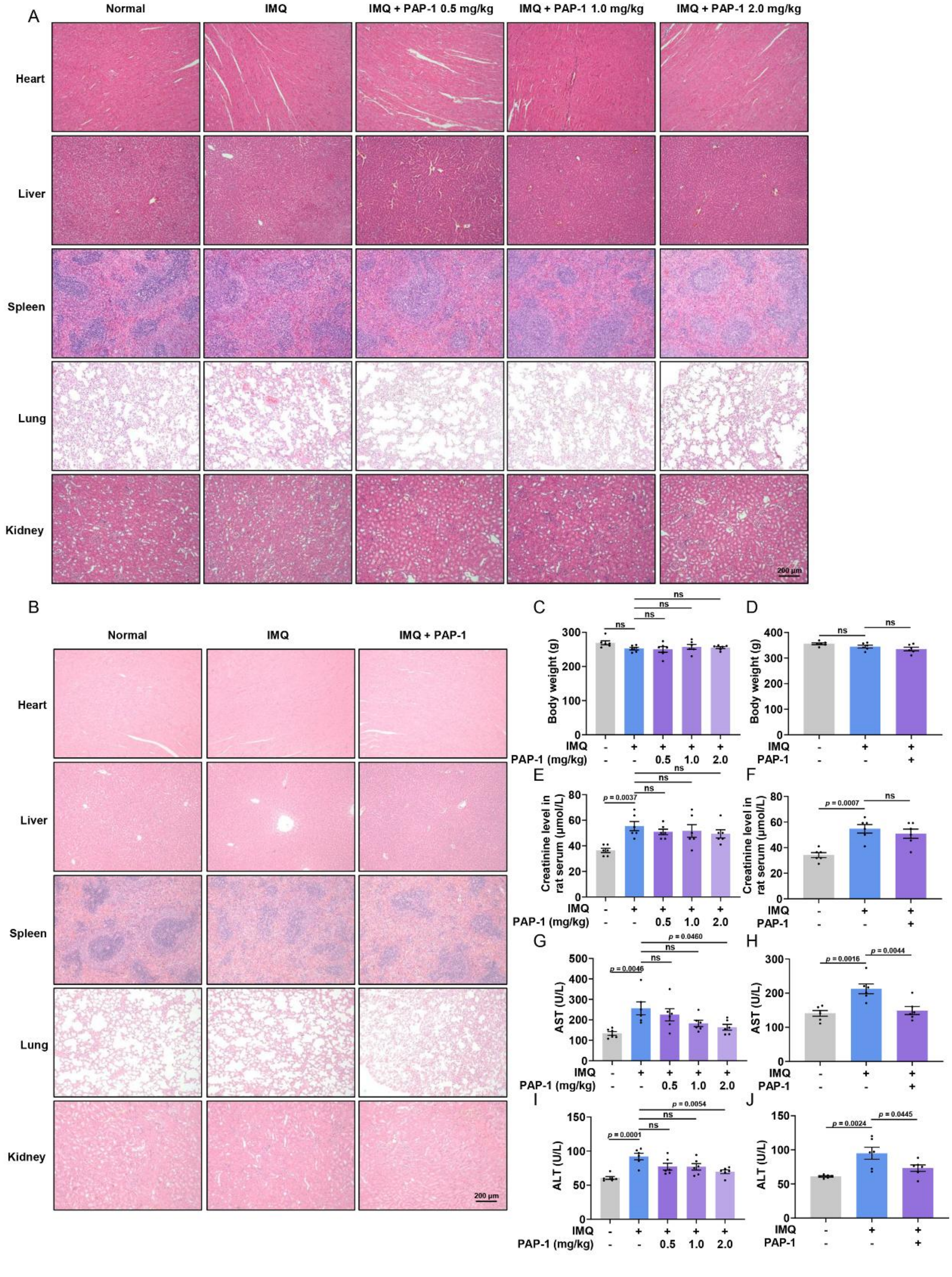

Supplementary Figure 1. Evaluation of the systemic safety of PAP-1 in male rats. (A and B) Representative H&E-stained sections of major organs (heart, liver, spleen, lung, kidney) from male rats receiving preventive (A) or therapeutic (B) PAP-1 treatment. Scale bar = 200 μm. (C and D) Body weight changes during the preventive (C) and therapeutic (D) intervention periods (n = 6). (E and F) Serum creatinine levels in the preventive (E) and therapeutic (F) treatment groups (n = 6). (G and H) Serum

AST levels in the preventive (G) and therapeutic (H) treatment groups (n = 6). (I and J) Serum ALT levels in the preventive (I) and therapeutic (J) treatment groups (n = 6). $p < 0.05$ was considered statistically significant; ns indicates non-significant.

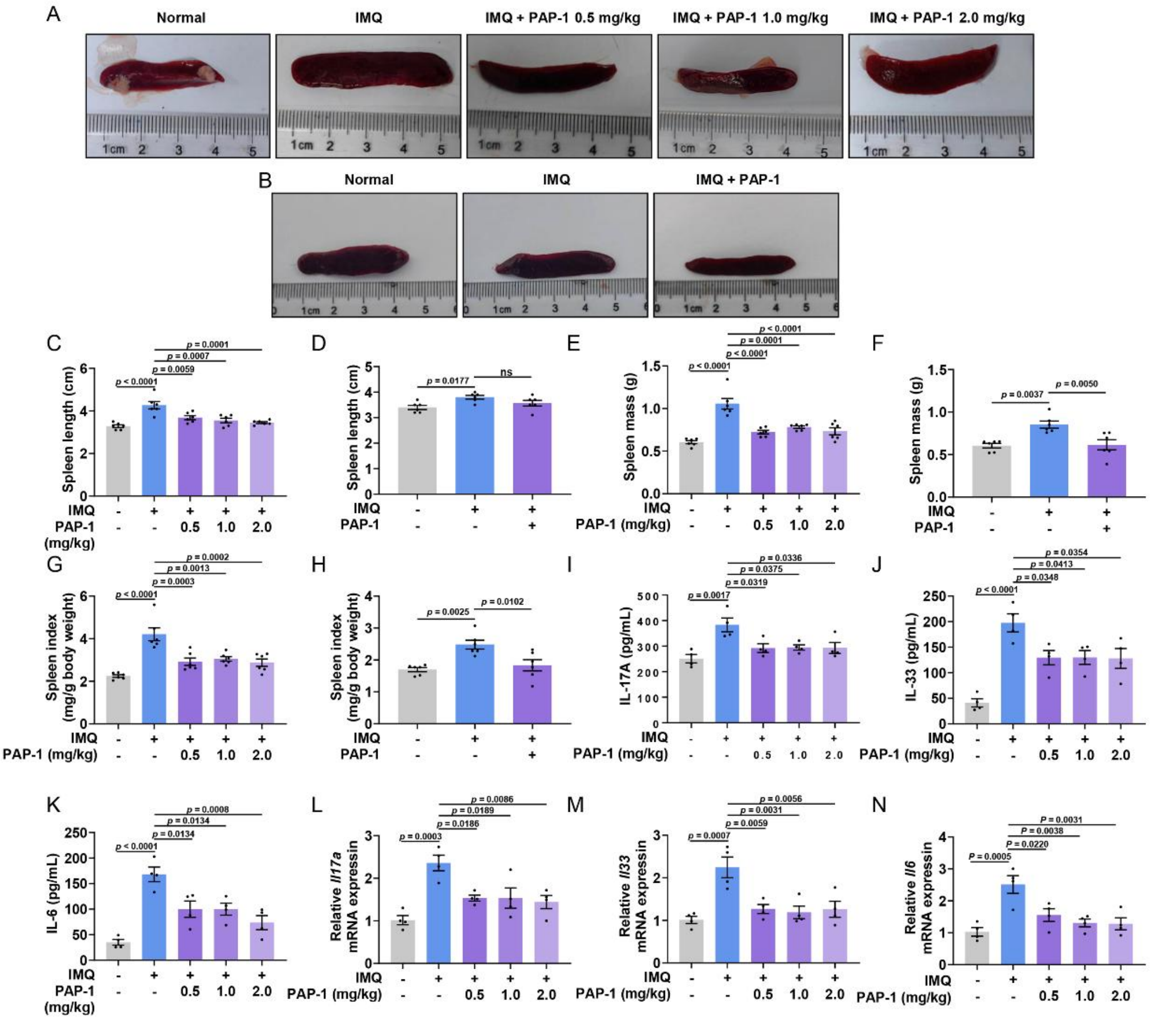

Supplementary Figure 2. PAP-1 mitigates inflammatory response in IMQ-induced psoriasis-like male rats. (**A and B**) Representative photographs of spleen in rats from the preventive (A) and therapeutic (B) groups. (**C-H)** Spleen length (**C and D**), spleen mass (**E and F**), and spleen index (**G and H**) in rats from the preventive (**C, E and G**) and therapeutic (**D, F and H**) groups (n = 6 rats/group). (**I, J and K**) Concentrations of IL-17A, IL-33, and IL-6 in rat serum were determined and quantified by ELISA (n = 4 rats/group). (**L, M and N**) mRNA expression levels of *Il17a*, *Il33*, and *Il6* in the affected dorsal skin of rats were measured by RT-PCR (n = 4 rats/group). Data are presented as mean ± SEM. $p < 0.05$ was considered statistically significant; ns indicates non-significant.

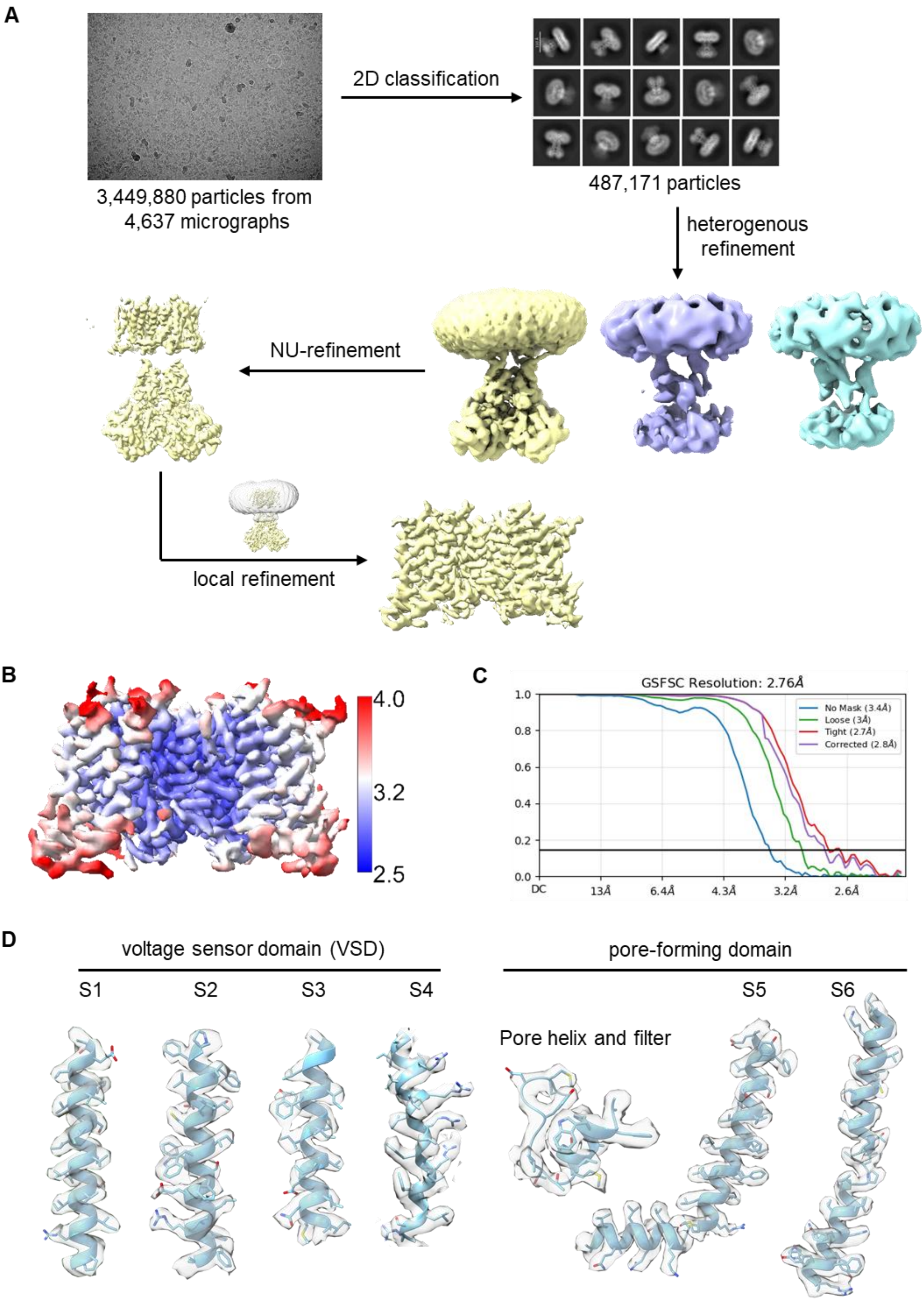

Supplementary Figure 3. Cryo-EM structure determination of the Kv1.3/PAP-1 complex. (**A**) Cryo-EM data processing flow chart. (**B**) EM density colored according to local resolution estimate. (**C**) Gold-standard Fourier shell correlation curves from CryoSPARC. (**D**) Cryo-EM density and fitted models of the transmembrane segments of Kv1.3.

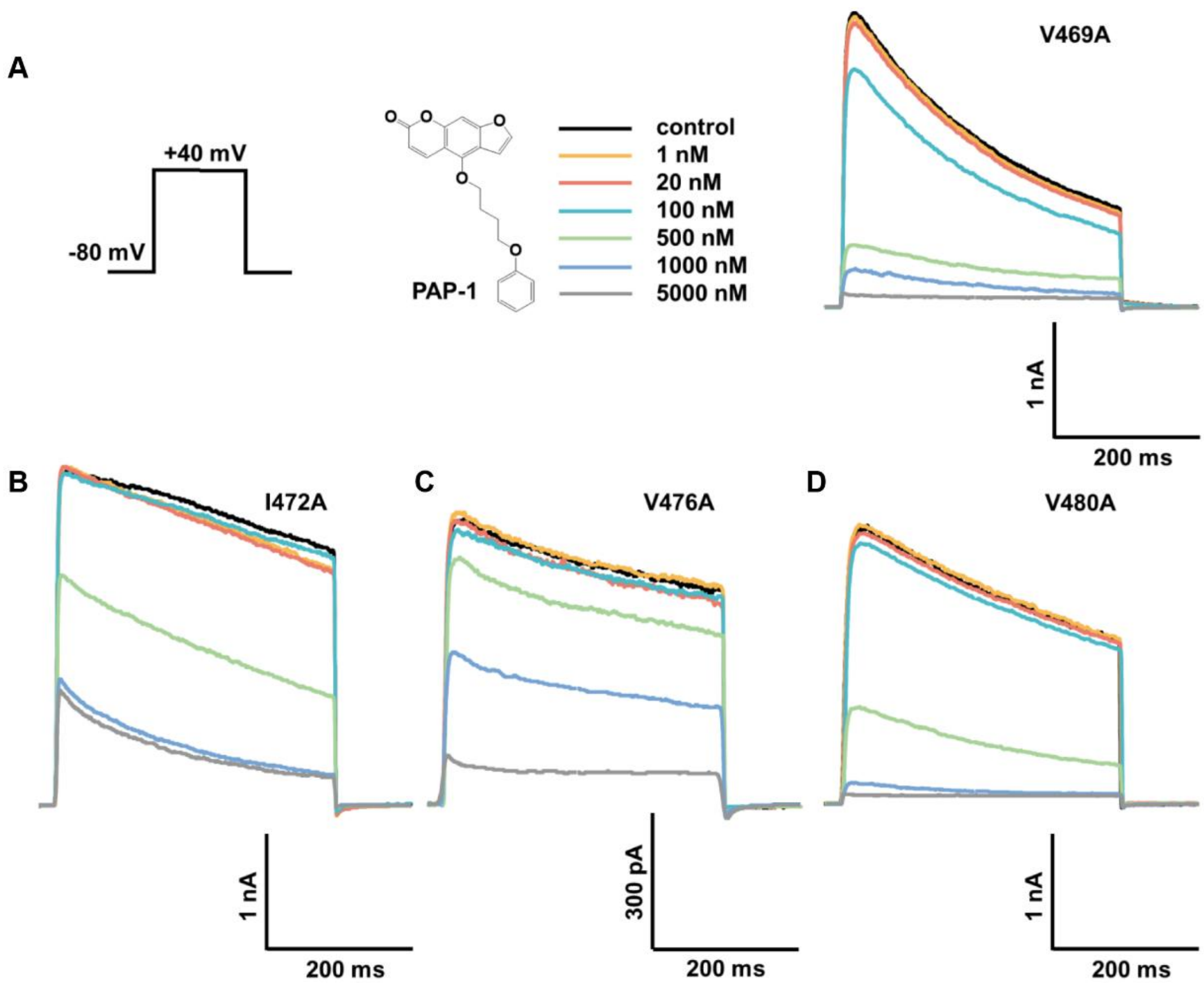

Supplementary Figure 4. Concentration-dependent inhibition of Kv1.3 mutants by PAP-1. (**A-D**) Representative whole-cell recording traces from CHO cells expressing Kv1.3 mutants (V469A, I472A, V476A, V480A), each showing concentration-dependent inhibition by PAP-1.

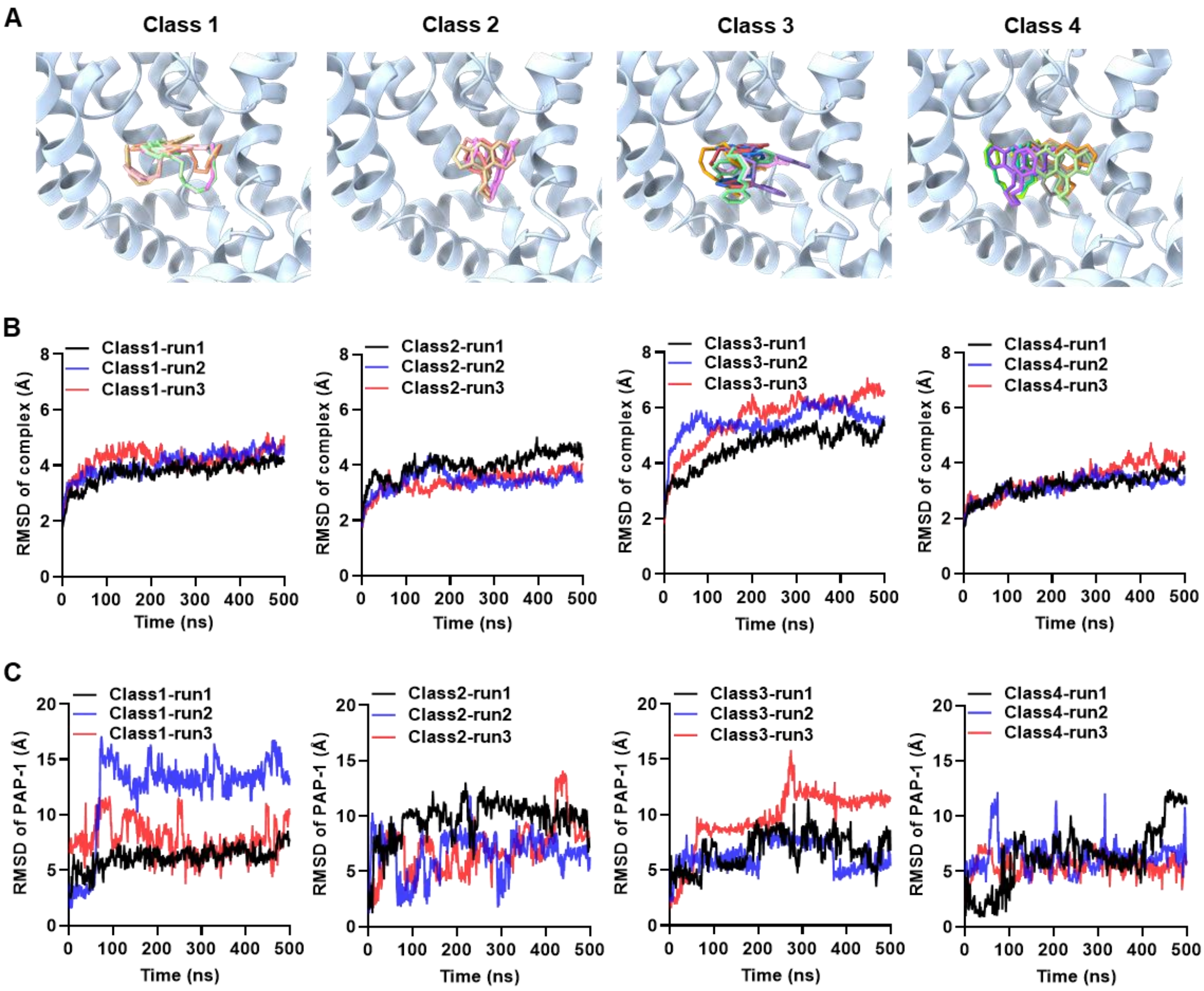

Supplementary Figure 5. Docking results for the Kv1.3/PAP-1 complex. (**A**) The top 30 docking-predicted binding conformations of PAP-1 were classified into four classes. (**B**) RMSD values for the Kv1.3/PAP-1 complex corresponding to different classes of PAP-1 structures. (**C**) RMSD values for the PAP-1 molecule in different classes of Kv1.3/PAP-1 structures.

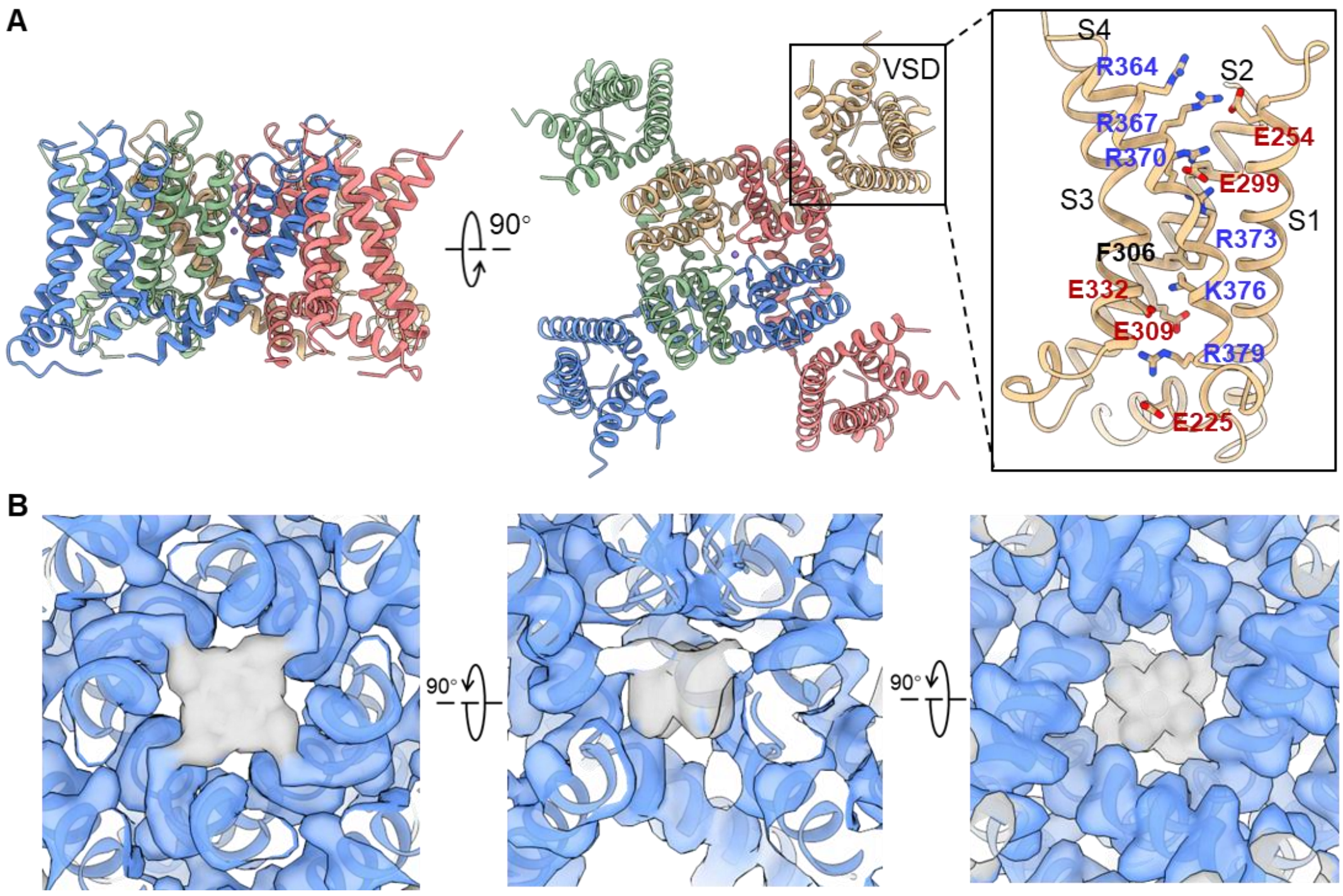

Supplementary Figure 6. Cryo-EM structure of the Kv1.3/PAP-1 complex. (**A**) The Kv1.3 TMD is shown as ribbons, with the VSD displayed in detail. Negatively and positively charged residues are colored red and blue, respectively. F306 separates the negatively charged residues located at the outer and inner ends of the VSD. The α-carbon of R373 is positioned at the level of F306. (**B**) Cryo-EM density (semi-transparent contoured, contoured at 4.5 σ) and fitted models of the Kv1.3 TMD, together with the potential density corresponding to PAP-1, viewed from the extracellular side (left), the membrane plane (middle), and the intracellular side (right).

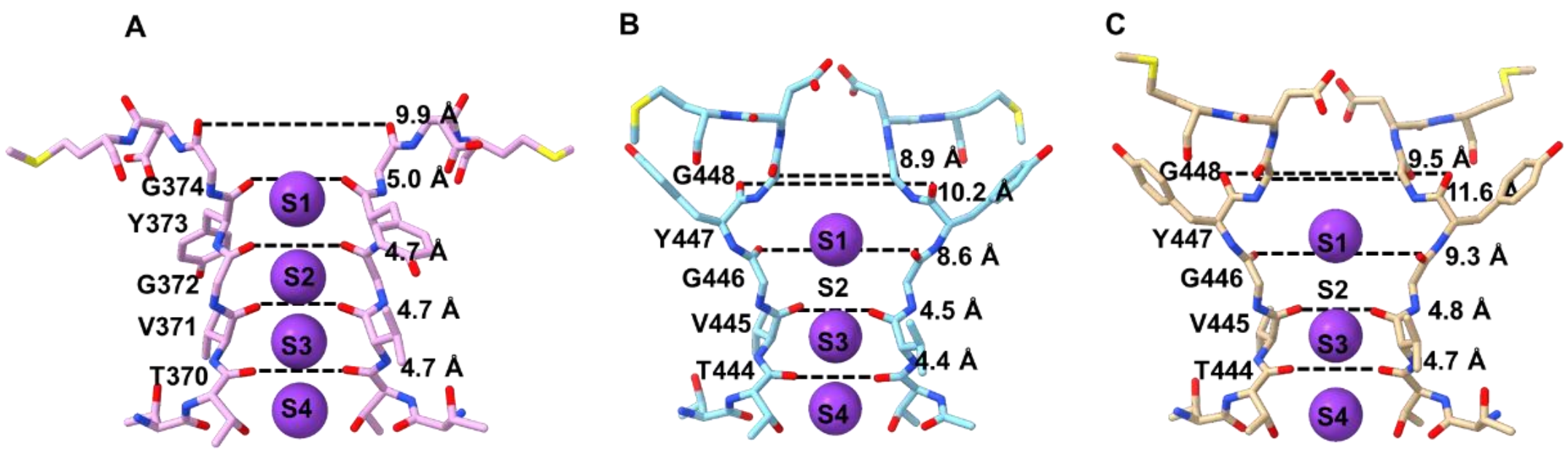

Supplementary Figure 7. Comparison of Kv channel selectivity filter conformations. (A-C) Side views of the selectivity filters from representative Kv channel structures. (**A**) Kv1.2-2.1 paddle chimera (PDB: 2R9R), representing a conducting selectivity filter conformation. (**B**) Kv1.3/PAP-1 complex. (**C**) Kv1.3-D2 conformation (PDB: 7SSY), representing an inactivated-like selectivity filter conformation. Selectivity filter residues are shown as sticks, potassium ion-binding sites as purple spheres labeled S1–S4, and measured distances between opposing TVGYG carbonyl oxygens as black dashed lines. The selectivity filter in the Kv1.3/PAP-1 structure displays a dilated architecture and reduced ion occupancy at the upper filter sites, differing from the conducting filter in Kv1.2-2.1. Instead, it closely resembles the Kv1.3-D2 filter conformation, suggesting that the PAP-1-bound Kv1.3 structure adopts an inactivated-like selectivity filter architecture.

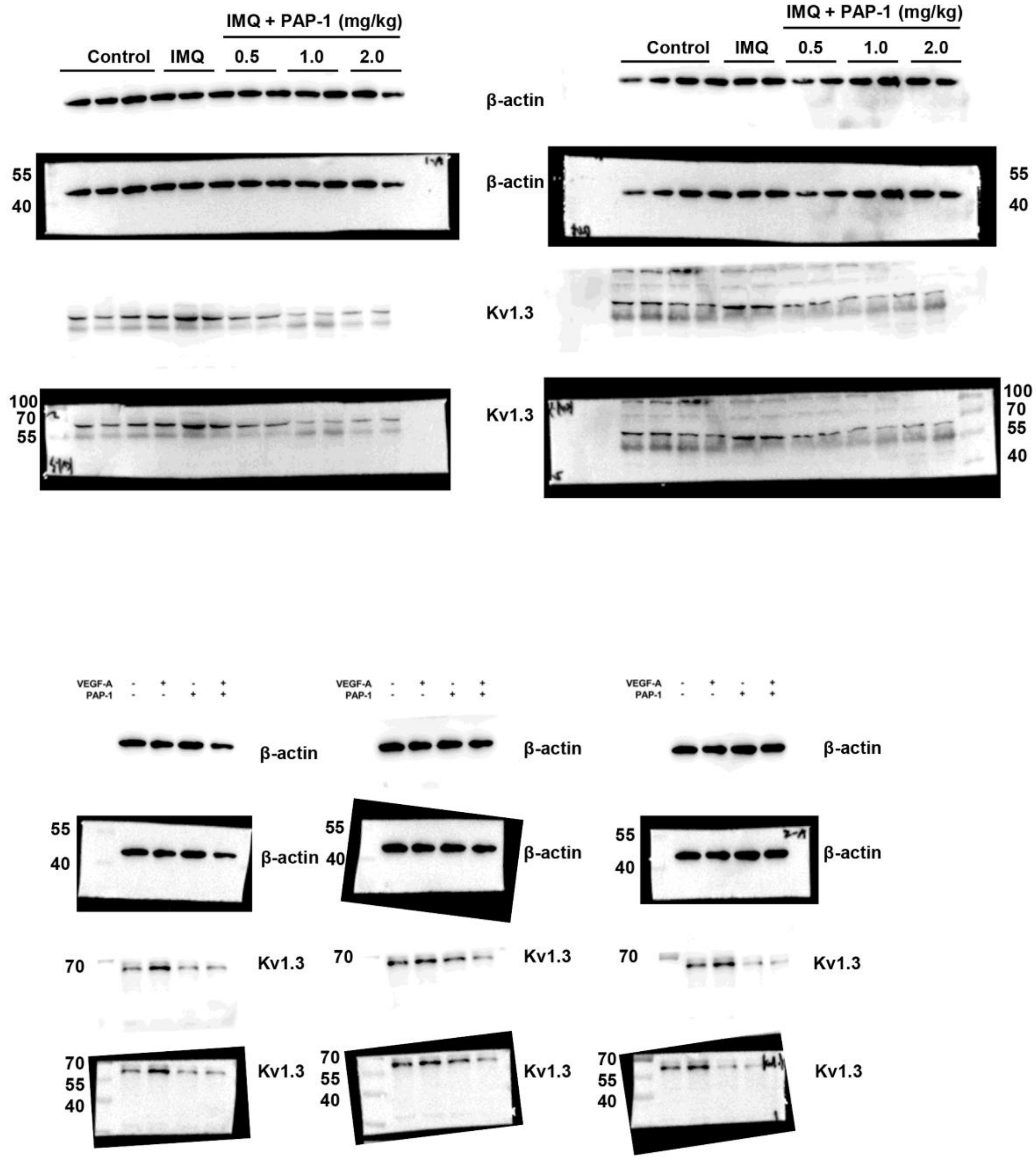

Supplementary Figure 8. Uncropped and unedited blot images.
