# Supplementary material for: The Kv1.3 blocker PAP‑1 alleviates psoriasis‑like dermatitis in rats via suppressing inflammation and angiogenesis
Source: Commun Biol. 2026 Jul 20;9:1497. doi: 10.1038/s42003-026-10718-7 (PMC13748653; doi:10.1038/s42003-026-10718-7)
Supplement: Supplementary file 2 [file 42003_2026_10718_MOESM2_ESM.pdf]

Description of Additional Supplementary Files

File name: Supplementary Data 1

Description: Supplementary Data 1 contains the initial coordinates, the simulation input files, and a coordinate file of the final output of molecular dynamics simulations.

File name: Supplementary Data 2

Description: Supplementary Data 2 provides the plasmid sequence and its corresponding map.
