# Supplementary material for: The Kv1.3 blocker PAP‑1 alleviates psoriasis‑like dermatitis in rats via suppressing inflammation and angiogenesis
Source: Commun Biol. 2026 Jul 20;9:1497. doi: 10.1038/s42003-026-10718-7 (PMC13748653; doi:10.1038/s42003-026-10718-7)

Kv1.3Δ52-Kvβ2.1（Protein expression plasmid map）

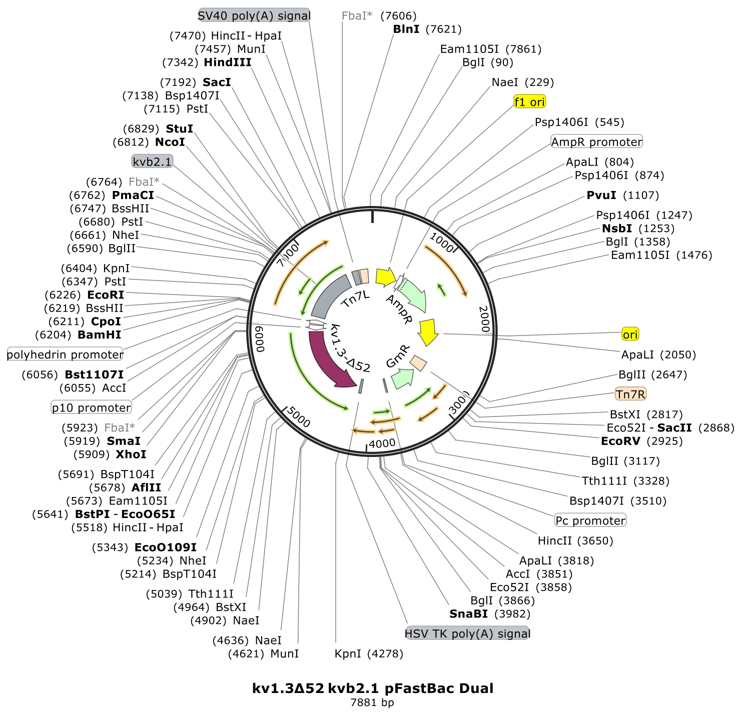

Kv1.3-WT（Electrophysiology plasmid map）

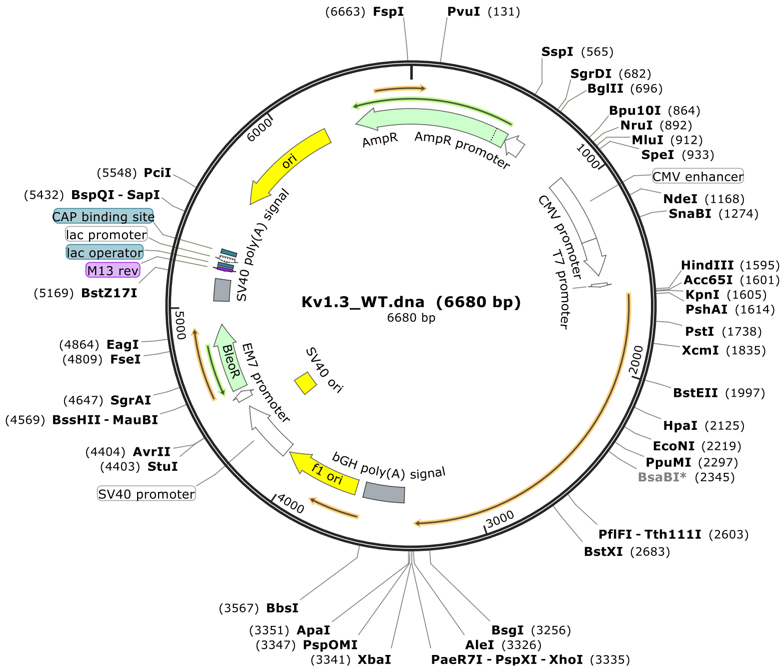

Kv1.3-V469A（Electrophysiology plasmid map）

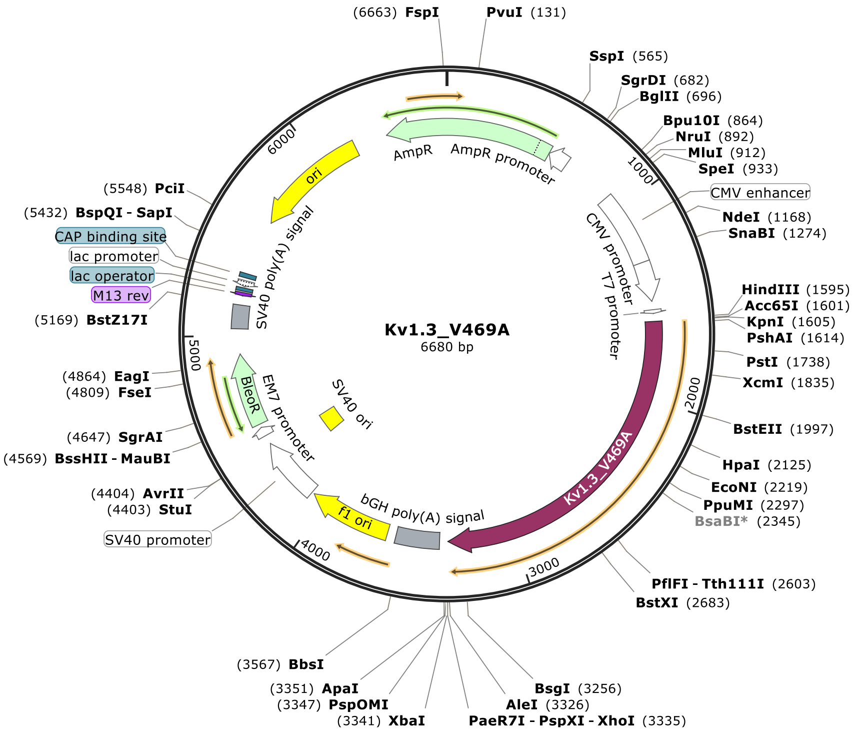

Kv1.3-I472A（Electrophysiology plasmid map）

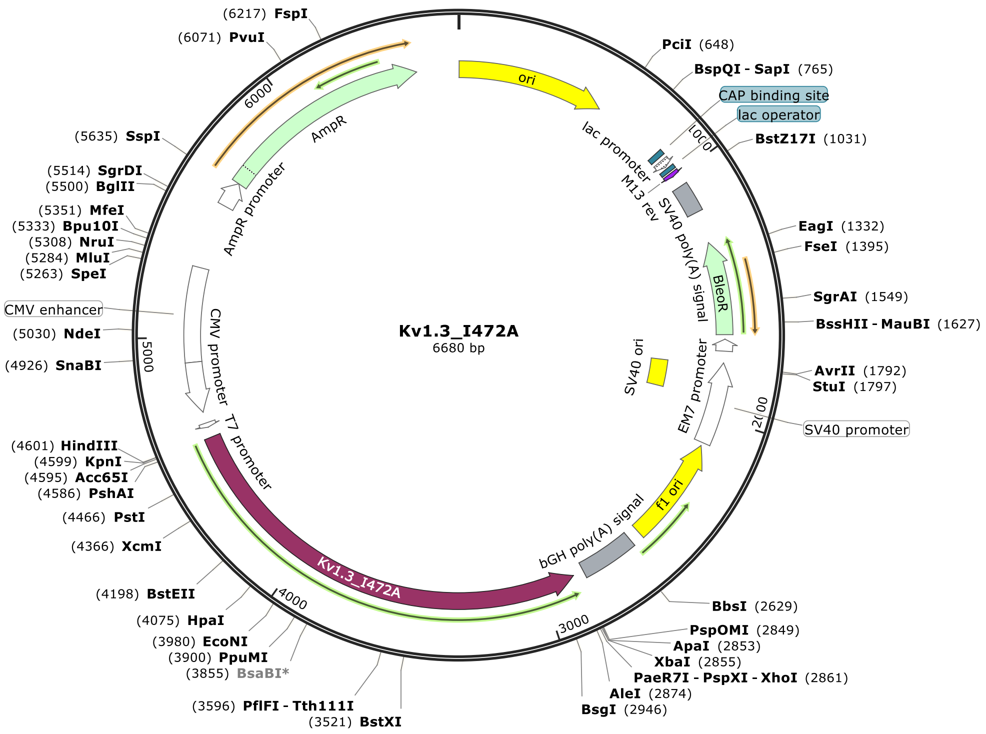

Kv1.3-V476A（Electrophysiology plasmid map）

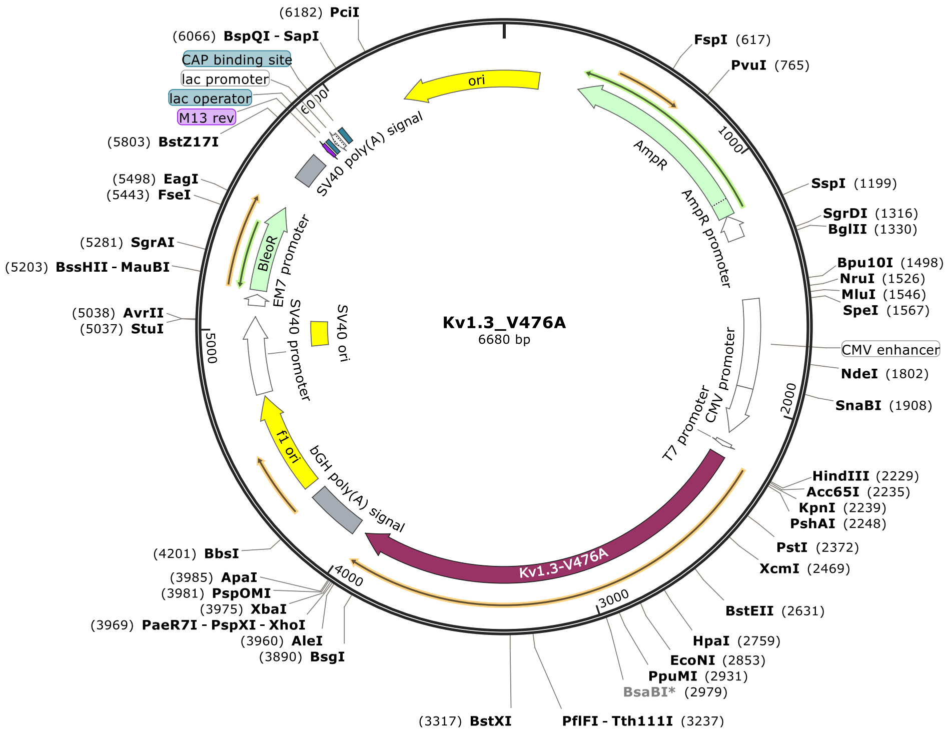

Kv1.3-V480A（Electrophysiology plasmid map）

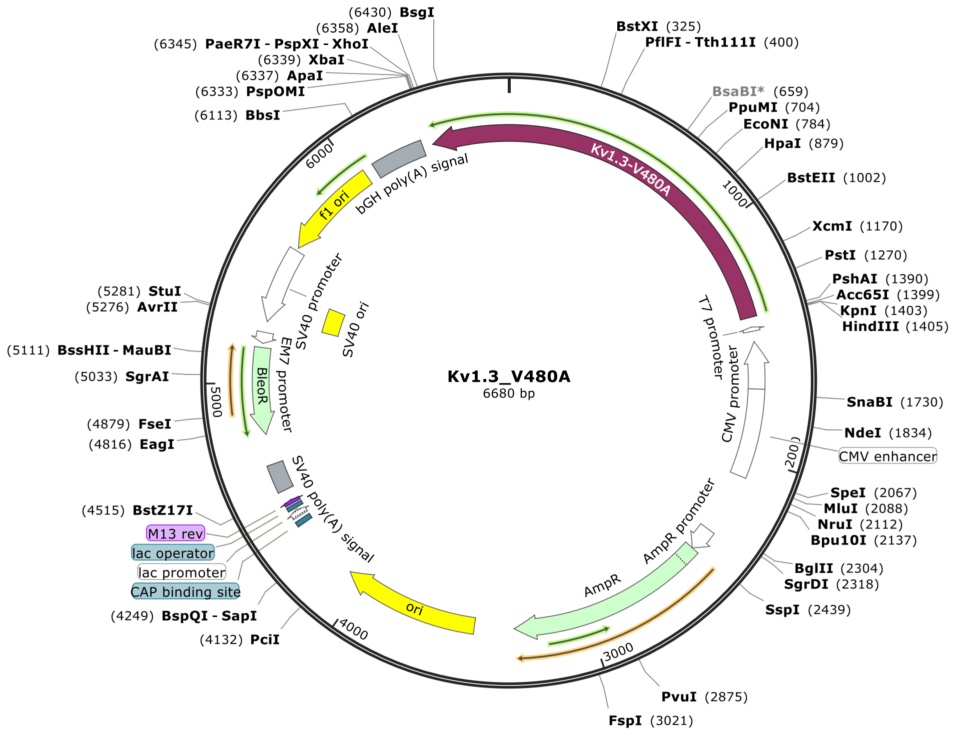

Supplement: Supplementary file 5 [file 42003_2026_10718_MOESM4_ESM.zip › Supplementary Data 2/Plasmid map.pdf]
